# Biocompatible Core–Shell Microneedle Sensor Filled with Zwitterionic Polymer Hydrogel for Rapid Continuous Transdermal Monitoring

**DOI:** 10.1021/acsnano.4c02997

**Published:** 2024-09-19

**Authors:** Shicheng Zhou, Yutaro Chino, Toshihiro Kasama, Ryo Miyake, Shigenobu Mitsuzawa, Yinan Luan, Norzahirah Binti Ahmad, Hiroshi Hibino, Madoka Takai

**Affiliations:** † Department of Bioengineering, The University of Tokyo, Tokyo 113-8654, Japan; ‡ Sanyo Chemical Industries, Ltd., Kyoto 605-0995, Japan; § Institute of Nano-Life-Systems, Institutes of Innovation for Future Society, Nagoya University, Nagoya 236-0027, Japan; ∥ Honda R&D Co., Ltd., Saitama 351-0193, Japan; ⊥ Division of Glocal Pharmacology, Department of Pharmacology, Graduate School of Medicine, Osaka University, Osaka 565-0871, Japan; # AMED-CREST, AMED, Osaka 565-0871, Japan

**Keywords:** polylactic acid, microneedles, electroless plating, electrochemical biosensors, zwitterionic polymer hydrogel, micro-nanofabrication

## Abstract

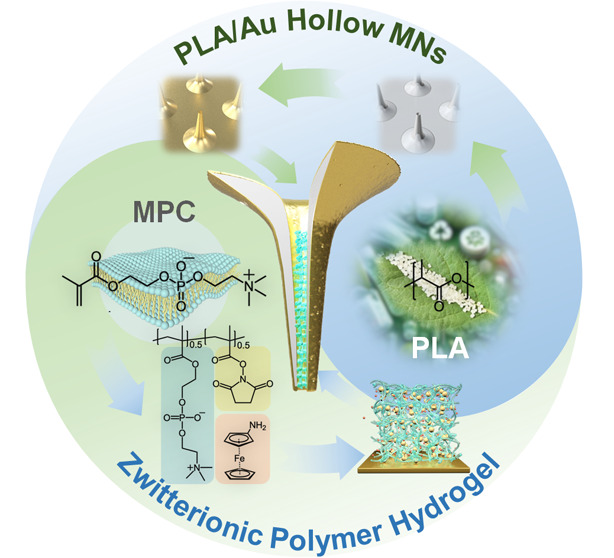

Microneedle (MN)-based electrochemical biosensors hold promising potential for noninvasive continuous monitoring of interstitial fluid biomarkers. However, challenges, such as instability and biofouling, exist. This study proposes a design employing hollow MN to encapsulate a zwitterionic polymer hydrogel sensing layer with excellent biocompatibility and antifouling properties to address these issues. MN shell isolates the internal microporous sensing layer from subcutaneous friction, and the hydrogel filling leverages the MNs’ three-dimensional structures, enabling high-dense loading of biorecognition elements. The hollow MNs are successfully fabricated from high-molecular-weight polylactic acid via drawing lithography, exhibiting sufficient strength for effective epidermis penetration. Additionally, a high-performance gold nanoconductive layer is successfully deposited inside the MN hollow channel, establishing a stable electrical connection between the polymer MN and the hydrogel sensing layer. To support the design, numerical simulations of position-based diffusive analyte solutes reveal fast-responsive electrochemical signals attributed to the high diffusion coefficient of the hydrogel and the concentrated structure of the hollow channel encapsulation. Experimental results and numerical simulations underscore the advantages of this design, showcasing rapid response, high sensitivity, long-term stability, and excellent antifouling properties. Fabricated MN sensors exhibited biosafety, feasibility, and effectiveness, with accurate and rapid in vivo glucose monitoring ability. This study emphasizes the significance of rational design, structural utilization, and micro-nanofabrication to unlock the untapped potential of MN biosensors.

Next-generation point-of-care devices, designed to be minimally or noninvasive, continuously measure clinically relevant biomarkers (e.g., glucose) in peripheral biofluids such as sweat, saliva, tears, and interstitial fluid (ISF).^1,2^ ISF, which closely reflects blood concentrations and facilitates comprehensive health assessment,^3,4^ is conveniently accessible near the skin’s surface and approximately three times more abundant than blood,^5^ offering a promising alternative for biomarker analysis. To syringe the advantages of ISF, microneedle (MN) technology has emerged as a crucial solution for accessing ISF and realizing real-time monitoring.^6,7^ Furthermore, it complements and surpasses commercial continuous glucose monitors by penetrating the dermis at a shallower depth, avoiding nerve endings and capillary beds, thus enabling a blood-free, pain-free, and minimally invasive approach.^8^ Compared to epidermal patch-type sensors, MN structure provides more design flexibility and analyte-accessing ability, enabling versatile applications, including ISF extraction,^9^ biorecepting,^10^ or transducing.^11^ MN-based enzymatic electrochemical sensors, with their high sensitivity, selectivity, and system integration maturity advantages, hold significant potential for commercialization.^2^

MN is typically designed with a tip diameter of approximately 50 μm and a length of 1000 μm, causing no local bleeding and minimal pain during insertion.^1,3,7^ As a sensor–skin interface tool, the MN must penetrate the epidermis effectively and robustly to maximize contact with the dermis and maintain its functional integrity after successful skin insertion. The common sensing format involves extracting ISF using hydrogel,^12,13^ hollow,^14,15^ and porous MNs^16,17^ toward back-loaded electrochemical electrodes outside the body. However, the limited fluid extraction capacity^18^ leads to the reliance on external accessories such as vacuum,^5^ pumps,^15^ pressure,^18^ or iontophoresis current,^19^ which increase device bulkiness and cause discomfort or pain.^20^ Another recently well-adopted practical sensor design assembles the sensing component on the tip, allowing direct contact of the sensors-embedded MN array with the dermal ISF for in situ, continuous acquisition of clinical data.^1,3^ Nevertheless, functionality loss, potential damage, and denaturation of biological recognition elements (e.g., enzymes) caused by epidermal insertion and subcutaneous friction can result in sensor deterioration and inaccuracies.^21,22^ Despite solutions being proposed, such as polymer protective coatings^23−25^ and extra embedded electrodes,^22,26−28^ these strategies increase analyte diffusion resistance, introduce fabrication complexity, and reduce the sensing area, failing to fully exploit the functional potential of MN structure.

As for the sensing layer’s construction, various methods are proposed to immobilize enzymes through electrostatic, covalent, and cross-linking techniques.^29,30^ Moreover, polymer hydrogel platforms have emerged as superior alternatives to traditional immobilization methods on two-dimensional surfaces.^31^ The water environment of hydrogel is suitable for preserving enzyme activity, constructing three-dimensional catalytic reaction pathways, and establishing biocompatible electrode–sample interfaces.^32^ Additionally, hydrogels can resist nonspecific adsorption through hydrophilic effects, protect biorecognition components from degradation, and enhance the stability of implanted electrochemical sensors.^33,34^ Despite these advantages, challenges emerge in hydrogel–electrode integration, miniaturization, and failure behaviors owing to hydration and weak mechanical properties during repeated use.^32,35−37^ Current MN sensor application scenarios include protecting the hydrogel with an additional inert polymer layer^38^ or directly separating the hydrogel from the enzyme layer as a sampling layer.^39^ These methods do not fully utilize the advantages of the hydrogel and are merely an extension of traditional 2D applications on the MN sensor. Notably, an intrinsically antifouling zwitterionic typed phosphorylcholine (PC) copolymer hydrogel was previously developed in our group, offering covalent immobilization as a postmodification property.^40^ We propose integrating this hydrogel with MN electrodes, and the resulting structure is anticipated to be an ideal candidate for transdermal electrochemical sensors. However, current subcutaneous MN designs expose the sensor directly to tissue friction, making it challenging to achieve reliable loading and long-term protection of the fragile hydrogel sensing layer. Consequently, the MN structure design and sensing layer integration scenarios require careful reconsideration to address these issues.

In this study, we propose an MN electrode design—a hydrogel-filled hollow MN electrode—for electrochemical glucose sensing (Figure 1), which can solve and utilize the swelling behavior of hydrogel to mechanically fix the sensing layer and utilize the advantages of hydrogel on tiny microneedle structures. Simultaneously, the three-dimensional volume of microneedles can also be fully utilized by hydrogels as excellent enzyme loading sites, ensuring that it is not limited to two-dimensional plane areas such as outer surfaces or needle tips, thus enhancing the sensing capabilities and increasing miniaturization—the combination of the hollow structure and the hydrogel sensing layer results in a synergistic effect. Various integrated techniques are proposed to realize this structure, including hollow MN fabrication, cyanide-free gold (Au) electroless plating, and the synthesis of zwitterionic hydrogel. The hollow MN matrix with the targeted 1000 μm length and 50 μm tip diameter, composed of biodegradable polylactic acid (PLA) metalized with Au, ensures biocompatibility and efficient skin insertion. Within the hollow channel, a PC-based copolymer hydrogel, covalently linked to a redox mediator and enzyme, serves as the electrochemical sensing layer. Furthermore, numerical simulations of this hydrogel-encapsulated hollow MN electrode, as a proof of concept, demonstrated the biosensor’s ability to rapidly detect an increase in ISF glucose concentration. These study findings will contribute to the advancement of minimally invasive transdermal devices, emphasizing the significance of this core–shell sensor structure design, delicate MN electrode assembling, and rational micro-nanofabrication technique integration to unlock the untapped potential of MN biosensors.

**Figure 1:**
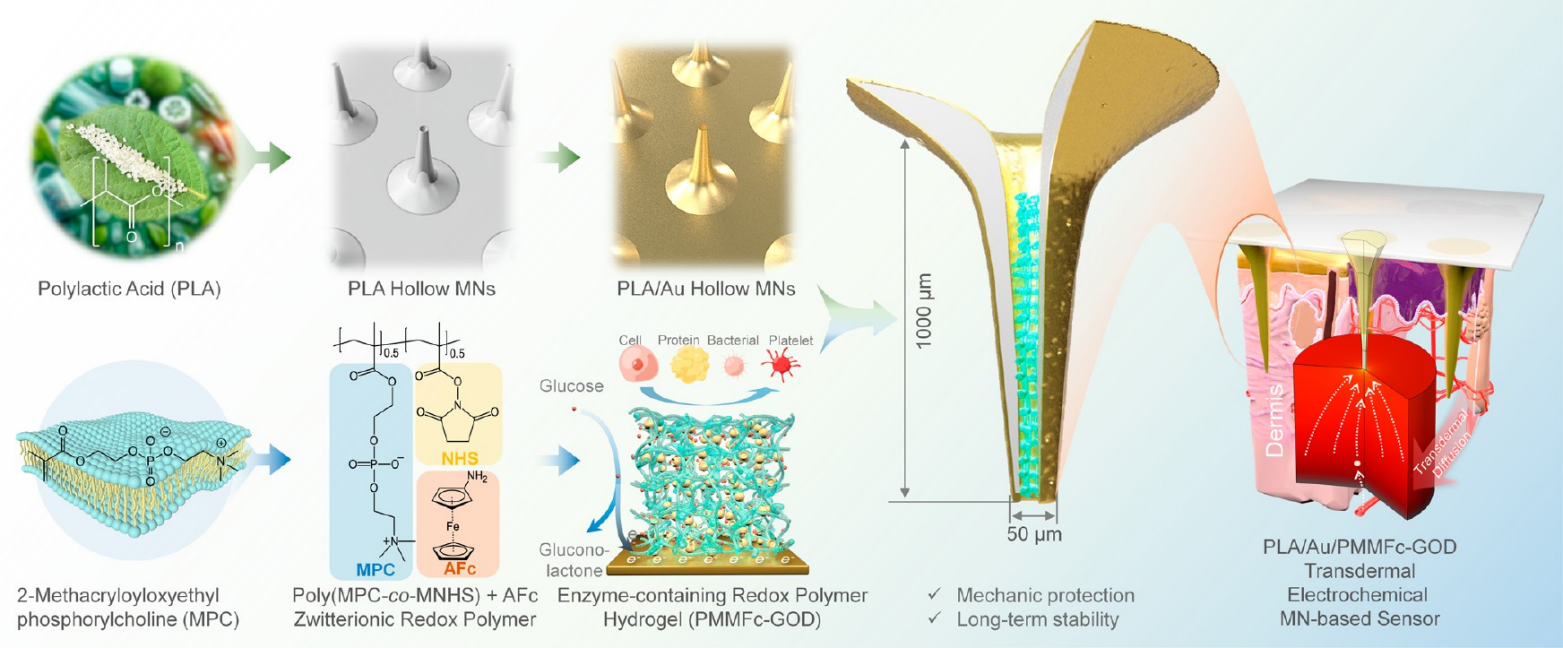
Schematic representation of the zwitterionic hydrogel-encapsulated PLA/Au hollow MNs and glucose diffusion during transdermal electrochemical sensing.

## Results and Discussion

### Fabrication of PLA Hollow MNs

**Figure 2:**
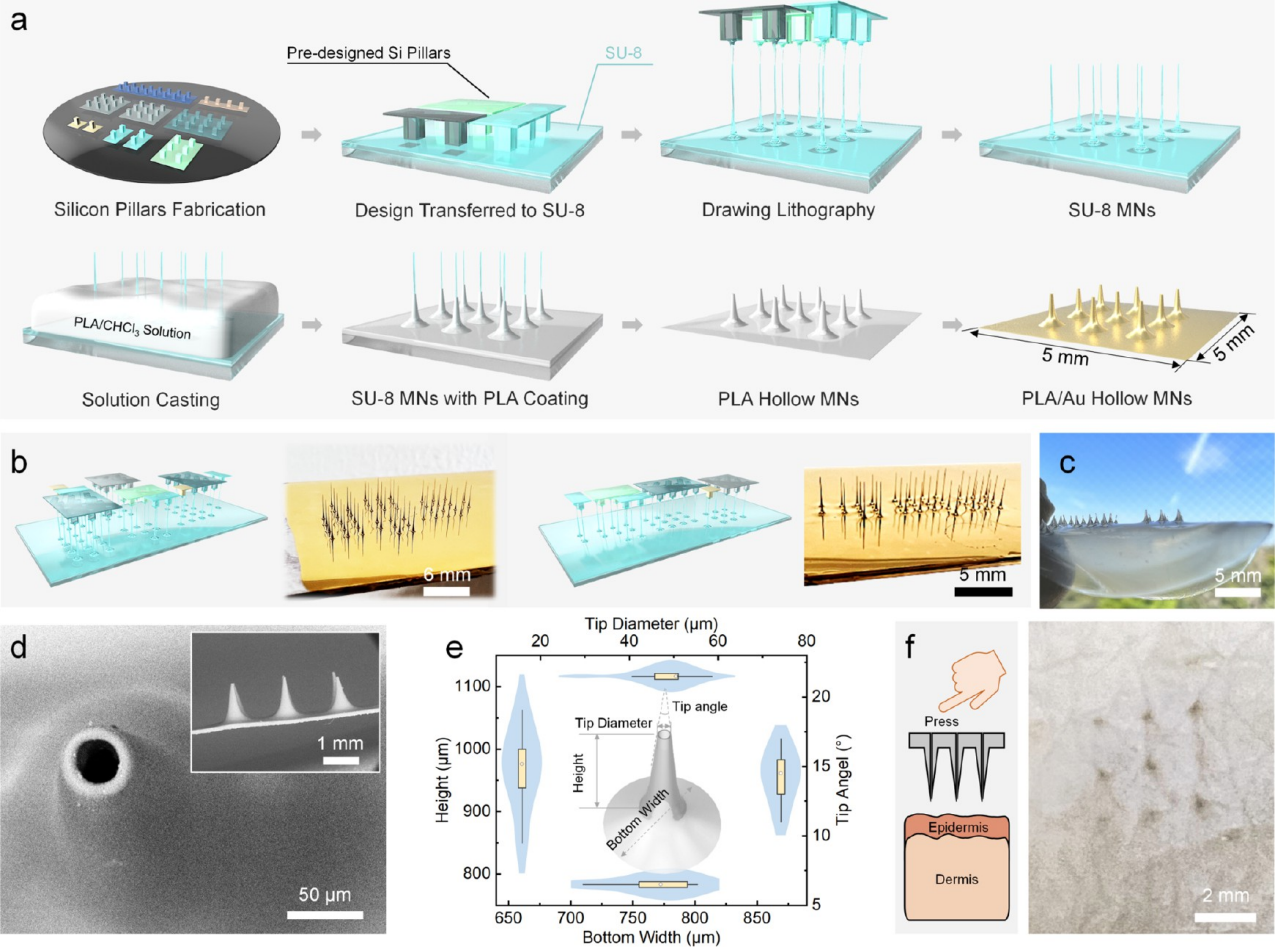
Fabrication of PLA hollow MNs. (a) Schematic representation of the fabrication procedures. (b) Structure transfer from the predesigned Si pillar to the SU-8 MN array. The electroless plating of Au on the SU-8 MNs aids in improving observability. (c) Optical and (d) SEM images of the fabricated PLA hollow MNs. (e) Summary of the MNs’ dimensions. (f) Insertion test of a 3 × 3 PLA hollow MN patch on porcine skin.

Fabricating MN with a balance of sharpness and strength presents significant challenges when dealing with high aspect ratio (AR) hollow designs, multineedle arrays, or specific configurations.^41^ Traditional methods, such as delicate silicon-based photolithography, involve costly micro-nanofabrication processes^42^ and raise health concerns regarding MN breakage and tip retention in the skin.^21^ Similarly, producing polymer/hydrogel MNs through micromachining or molding lacks precision or requires intricate multistep procedures. To address these issues, we propose an approach that combines drawing lithography with a one-step casting method to fabricate hollow MNs using biocompatible, high-molecular-weight (HMW) PLA (Figure 2a).

Drawing lithography was utilized to fabricate ultrahigh AR SU-8 MNs of various designs (Figures 2b and S1). Within the Si pillar size range of 300–1000 μm at drawing speeds of 25 and 50 μm s^–1^, SU-8 MN with high molding rates and consistency can be obtained in this broad processing window (Figure S2). By reducing the Si pillar size and adopting a relatively high drawing speed, the decreased MN diameter can be realized to ensure sharpness. The subsequent application of a PLA/CHCl_3_ solution spontaneously forms a dense PLA coating on the SU-8 MNs’ surface upon solvent evaporation. Carefully peeling off the PLA coating from the SU-8 MNs results in a freestanding patch of PLA hollow MNs. This strategy guarantees a simplified process and the ability to adjust the structure by controlling the solvent evaporation through the temperature. At lower temperatures, thinner tips and smoother substrates are expected to be formed, while thicker diameters along with rougher surfaces can be achieved at higher temperatures (Figure S3). This PLA hollow MN array patch, with an appearance of good transparency, provides ample space for Au metallization and sensing layer encapsulation (Figure 2c). Scanning electron microscopy (SEM) observation displays the MN structure with a length of approximately 1000 μm and a tip diameter of 50 μm (Figure 2d), featuring detailed dimensional features depicted in Figure 2e. This method can conveniently achieve the conical MN, offering distinct advantages, including reduced material usage^43^ and efficient skin penetration with minimal displacement.^44^ The skin insertion test confirms the successful penetration of the microneedles into porcine skin, creating visible micropores under fingertip pressure, which validates the reliable skin insertion performance of PLA hollow microneedles (Figure 2f).

### Au Metallization of PLA MN Patch

**Figure 3:**
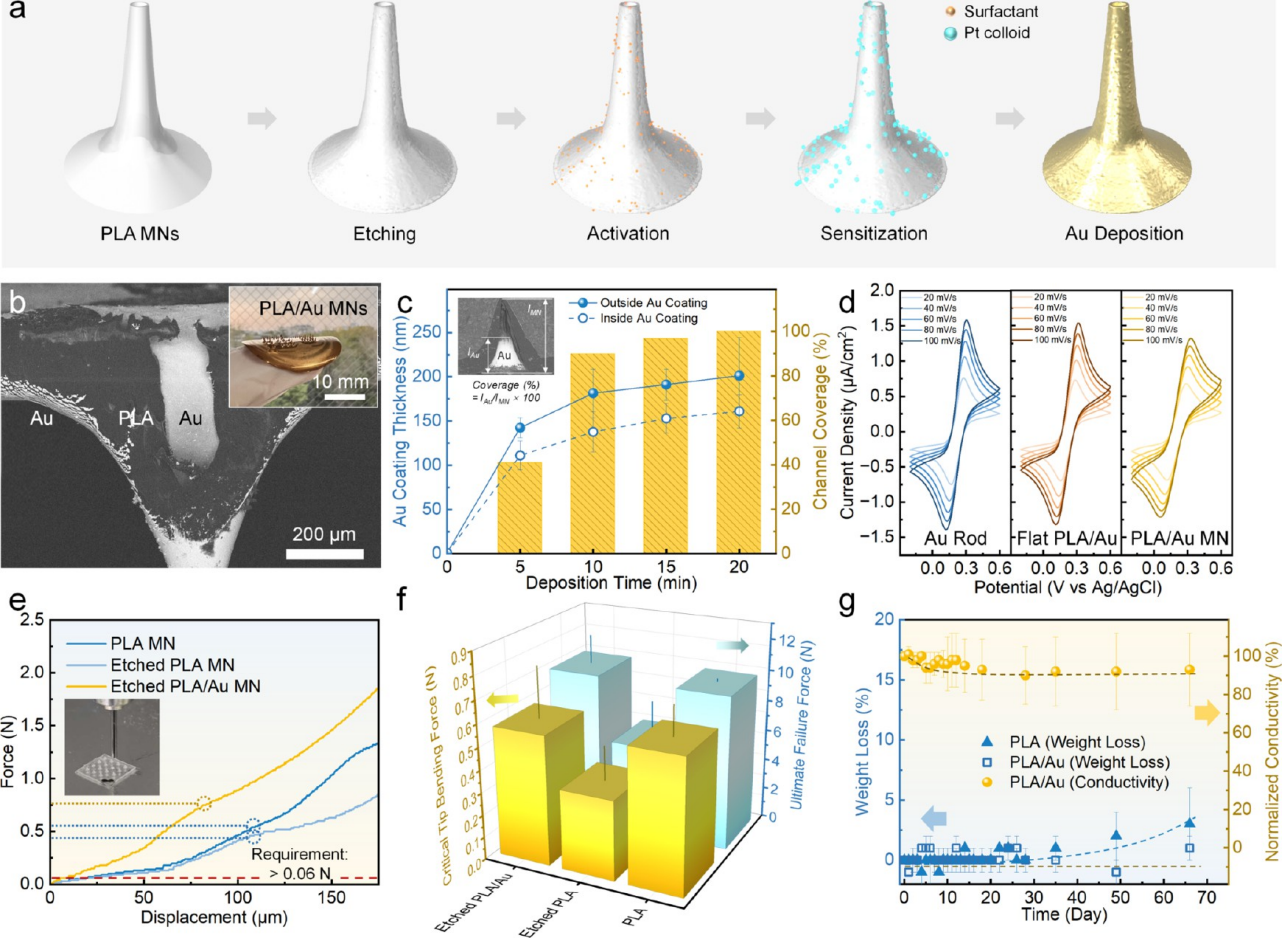
Au metallization on PLA hollow MNs. (a) Schematics of the cyanide-free electroless plating of Au on PLA. (b) SEM and optical images of deposited Au on PLA. (c) Relationship between the thickness and the deposition time. (d) Electrochemical evaluation of the PLA/Au electrodes in PBS with 10 mM Fe(CN)_6_^3–/4–^. (e) Force–displacement curves of PLA, etched PLA, and etched PLA/Au MNs obtained from the single-MN compression experiment. (f) Summary of the critical tip bending force and ultimate failure force. (g) Long-term degradation and stability evaluation of the PLA and PLA/Au electrodes immersed in artificial ISF at 37 °C.

Following the fabrication of hollow PLA MNs, the subsequent steps of metallization and circuitization are crucial for their application as electrodes. In most instances, physical vapor deposition (PVD), particularly sputtering, is commonly adopted for MN metallization. Despite its high efficiency, this method has several drawbacks, including high equipment costs, the necessity for an additional adhesive layer, and reduced ductility owing to the formation of nanograin boundaries.^45^ Moreover, sputtering can result in shadowing effects when depositing on structures with a depth-to-width AR greater than 1, leading to suboptimal sidewall coverage.^46^ Owing to our objective to decorate the inner channel of the hollow MNs with an AR of approximately 20, a cyanide-free electroless plating method was utilized (Figure 3a). The PLA was initially pretreated using a positively charged surfactant and sensitized using a platinum colloid to facilitate catalytic Au reductive deposition, as shown in eq 1
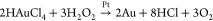
1This Pt-catalyzed Au reduction deposition can readily form smooth and continuous Au coatings on the PLA at room temperature with high electrical conductivity and flexibility (Figures 3b, S4, and S5a). The deposited Au layer has a thickness of approximately 150–200 nm, and the thickness does not increase significantly with prolonged deposition time as Au covers the catalytic Pt colloids (Figure S5b). This feature is consistent when deposited on both the inner and outer surfaces of hollow PLA MNs (Figure 3c). Furthermore, the channel coverage markedly increases to over 90% after electroless plating for 10 min. The Au coverage of the inside channel can also be confirmed by the formation of conductive paths on both surfaces using a multimeter.

Subsequently, an annealing process performed at temperatures above 150 °C significantly enhances the direct adhesion force between the Au layer and PLA patch (Figure S5c). A similar phenomenon was also observed in other polymers and Au after annealing.^47^ The selected annealing temperature, between the glass transition temperature and the melting point of PLA, affects the relaxation of molecular chains^48^ and causes a micronano deformation, contributing to the stable connection between the PLA and the nanostructured Au layer. Additionally, this annealing may aid PLA in transitioning from the less ordered α′ crystal form to the better ordered α crystal form, thereby enhancing the overall mechanical strength.^49^ The strong bond between the PLA and Au layers helps construct a smooth and continuous interface verified through cross-sectional SEM images and element distribution (Figure S5d–e).

The PLA/Au electrode also demonstrated an electrochemical performance comparable to that of commercially available standard Au rod electrodes (Figure 3d). The relationship between the peak current density and scan rate revealed a roughness factor of 1.04, representing the ratio of the electrochemical surface area to the geometric area for PLA/Au electrodes (Figure S6). To understand the mechanical properties of MN, detailed force–displacement curves, critical tip bending forces, and ultimate failure forces of PLA, etched PLA, and etched PLA/Au MNs were obtained from a single-MN compression test (Figure 3e,f). The critical bending force, where the tip becomes susceptible to compression and loses sharpness, was identified as the first force mutation point.^50^ Exceeding this threshold leads to tip bending and unsuccessful penetration. The tip bending and ultimate failure forces were not significantly reduced after the etching process compared to the as-prepared PLA MNs, despite improvement after Au deposition. All three types of microneedles far exceeded the requirements of 0.06 N for skin penetration.^51^ Etched PLA/Au microneedles exhibited an ultimate failure force of approximately 10 N, comparable to fused 3D-printed solid PLA microneedles.^52^

HMW PLA is widely used in long-term medical applications due to its superior mechanical properties and slow biodegradation, which extends up to 2–3 years inside the human body.^53^ The PLA/Au electrodes made of HMW PLA demonstrated excellent stability against biodegradation and subcutaneous friction, maintaining their structural integrity and retaining approximately 90% of their electrical conductivity after a 9-week immersion in artificial ISF and 50 skin insertions (Figures 3g and S5f). The decrease in conductivity can be attributed to minor coating cracks caused by repeated probe contact during measurements. The bare PLA electrodes exhibited a minor mass loss during the 5–9 weeks, which ensures the maintenance of structural integrity even in the event of an accidental detachment of the external Au layer during sensor application. The dual functionality of the outer Au layer, acting as both a protective coating and a PLA degradation regulator, addresses tip retention concerns. In cases of needle breakage within the subcutaneous tissue, where PLA is exposed without the protective Au coating, it undergoes accelerated degradation and is eventually hydrolyzed into lactic acid, a compound safely absorbed by the human body.

### Construction of Enzyme-Containing Redox Zwitterionic Polymer Hydrogel

**Figure 4:**
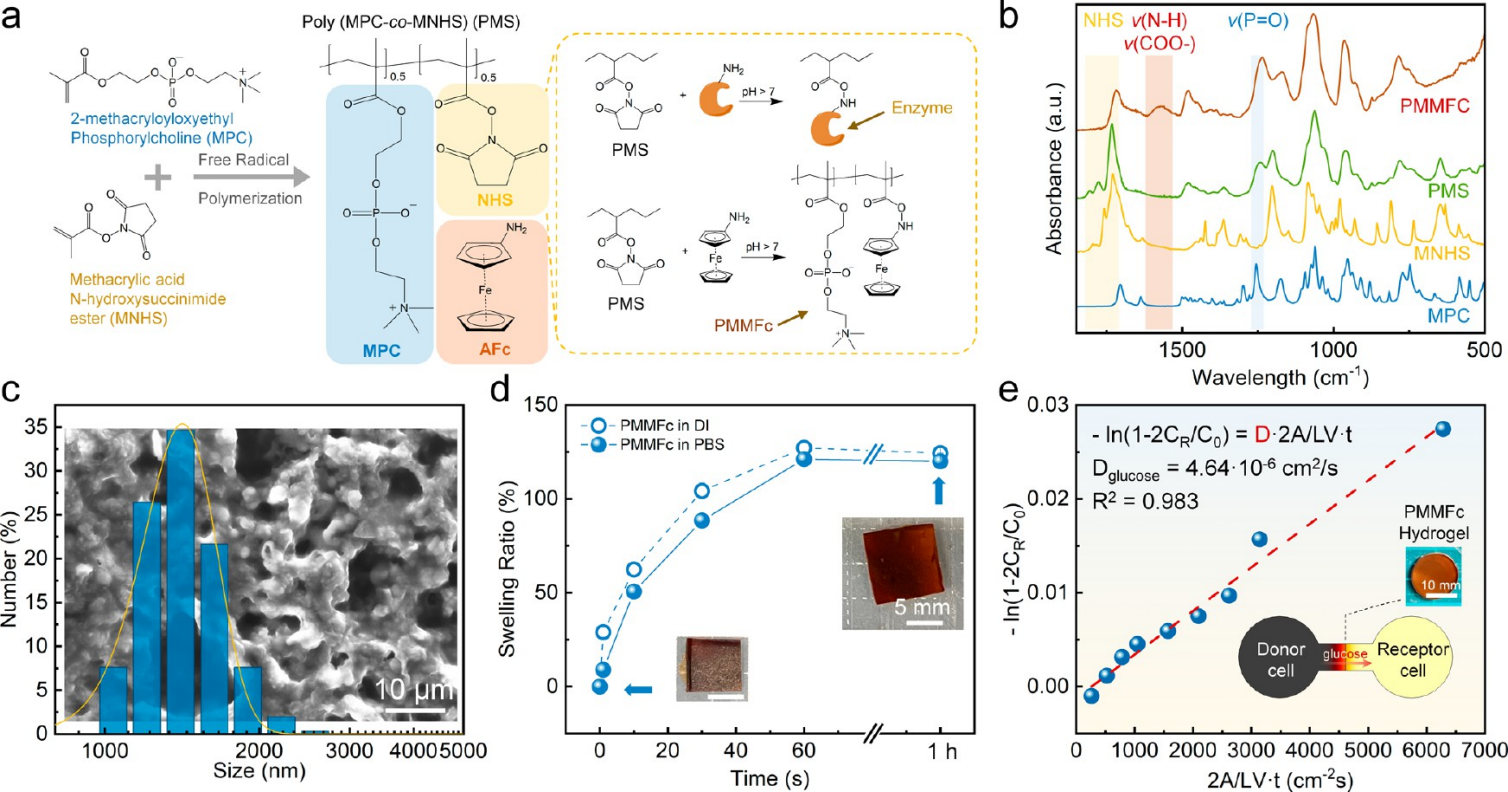
Synthesis and characterization of the zwitterionic redox hydrogel. (a) Schematic of the synthesis route and NHS-based covalent immobilization property of PMS. (b) FT-IR analysis of MPC, MNHS, PMS, and PMMFc. (c) SEM images, DLS evaluation, and (d) swelling behavior of the PMMFc-GOD hydrogel. (e) Diffusion coefficient of glucose in the PMMFc-GOD (denatured) hydrogel. The inserted model illustrates a simulation of a side-by-side diffusion Franz cell experiment.

The sensing layers’ construction must contend with challenges, including interface failure,^10^ biomolecule leakage (e.g., enzymes, redox mediators),^30^ and nonspecific adsorption.^54^ Existing strategies include modifications with poly(ethylene glycol) (PEG),^38^ hydroxyethyl methacrylate (HEMA),^55^ and zwitterionic polymer.^56−58^ The PC-based copolymers are the first-tier antifouling candidates owing to their strong interactions between interfacial water molecules.^59^ To leverage the advantages of zwitterionic polymers and address challenges related to biofouling and electrode stability, we designed an intrinsically antifouling zwitterionic polymer hydrogel and aimed to decorate it on PLA/Au electrodes. Figure 4a illustrates the synthesis of the zwitterionic PC-based copolymer hydrogel poly(MPC-*co*-MNHS) (PMS) and the subsequent procedures for the covalent immobilization of biomolecules. PMS is able to couple with amino ferrocene (AFc) via NHS-ester under alkaline conditions to form the redox polymer, which is denoted as PMMFc.

Figure 4b shows the FT-IR analysis of MPC, MNHS, PMS, and PMMFc, with the assigned characteristic bands in Table S1. The signal observed at 1255 cm^–1^ corresponds to the P=O stretch of the MPC phosphonic acid groups,^60,61^ which remained after the polymerization and amine coupling. Additionally, the triplex bands detected at 1732, 1777, and 1805 cm^–1^, originating from MNHS and PMS, are associated with the antisymmetric C=O, symmetric C=O, and carbonyl stretching bands of the NHS-ester, respectively.^62^ Upon reaction with AFc, the characteristic peaks of NHS-ester disappear in PMMFc. Consequently, a broad peak ranging from 1525 to 1615 cm^–1^ arises from the combination of the N-H bending band^63^ from coupled AFc and the O–C=O antisymmetric stretching band^64^ resulting from hydrolyzed NHS-ester. The zwitterionic MPC chains, known for their high affinity for water molecules, shield the hydrophobic NHS groups from water vapor, enhancing the long-term storage stability (Figure S7a).

Following the immobilization of glucose oxidase (GOD), the PMMFc-GOD hydrogel exhibited a microparticle network with a size distribution ranging from 1–2 μm and an average size of 1.5 μm from the SEM image and DLS results (Figure 4c). This hydrogel, featuring zwitterionic polymer groups and microporous structures, exhibits excellent diffusion efficiency for target analytes and antifouling properties. Additionally, owing to the hydrophilicity of the zwitterionic component, this hydrogel rapidly swells to its equilibrium state within 1 min in phosphate-buffered saline (PBS) buffer and deionized water (Figure 4d). The stability of the PMMFc hydrogel is further confirmed by cyclic swelling and deswelling tests (Figure S7b). To understand the diffusivity of glucose in PMMFc-GOD, whose enzyme has been denatured at 90 °C for 1 h, we measured its diffusion coefficient using a side-by-side Franz diffusion cell (Figure S8). The glucose concentration was recorded in the receptor cell over time, and the diffusion coefficient was calculated to be 4.64 × 10^–6^ cm^2^ s^–1^, which was validated using a numerical simulation (Figures 4e and S8c).

### Construction of PLA/Au/PMMFc-GOD Flat Sensor

**Figure 5:**
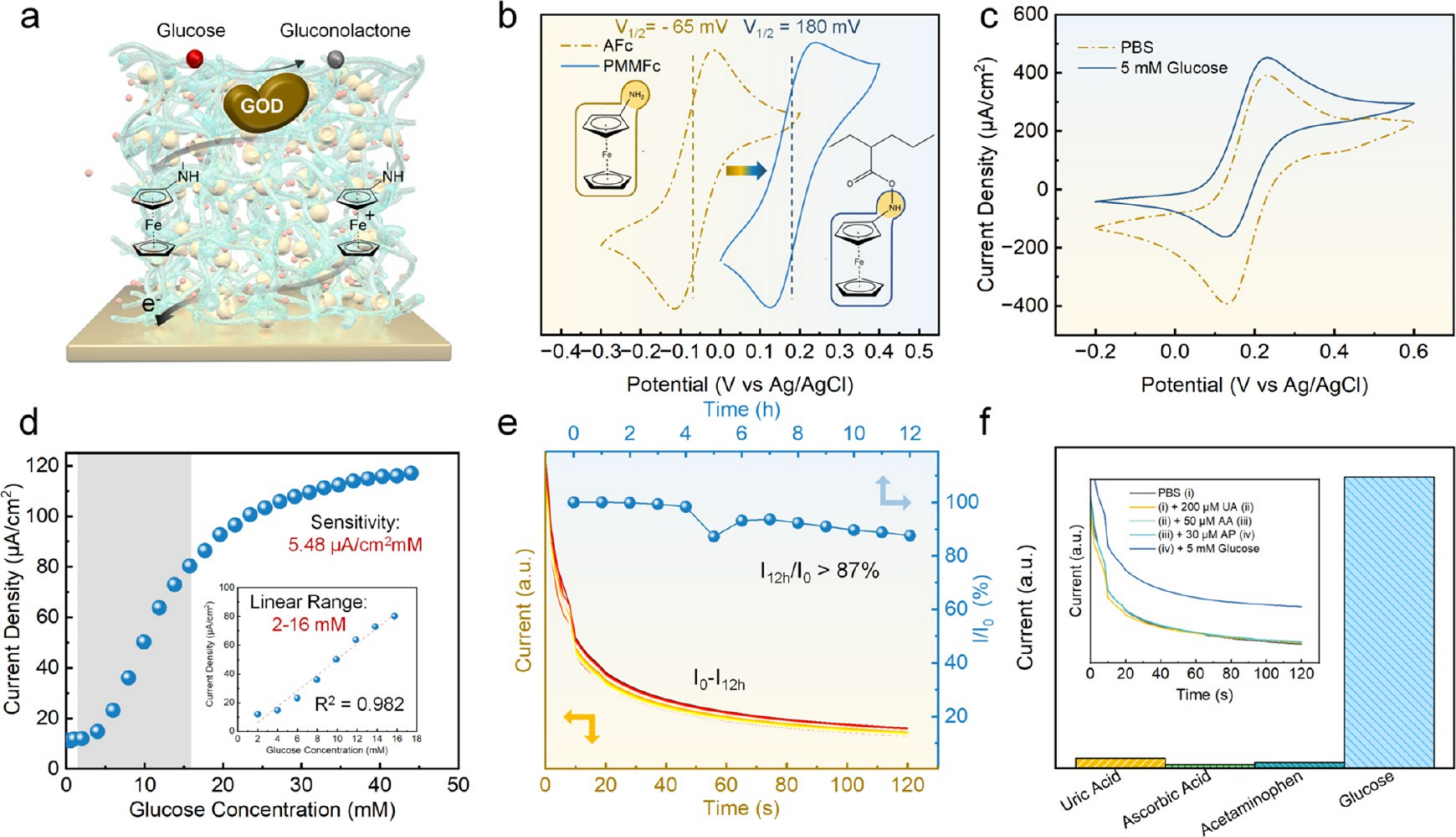
Electrochemical performance of the PLA/Au/PMMFc-GOD flat sensor. (a) Schematic of the PMMFc-mediated enzymatic electrochemical reaction of the glucose sensor. (b) Redox potential shift of PMMFc owing to covalent immobilization of ferrocene moiety. (c) CV results of glucose sensing behavior, (d) glucose calibration curve from the chronoamperometric curve at +0.24 V, (e) stability during continuous measurement for 12 h, and (f) selectivity evaluation against UA, AA, and AP of the PLA/Au/PMMFc-GOD flat sensor.

Through the simple drop-casting of the PMMFc-GOD sol on flat PLA/Au electrodes, a PLA/Au/PMMFc-GOD flat sensor can be constructed. The anticipated electrochemical enzymatic reaction in PLA/Au/PMMFc-GOD is shown in Figure 5a and eqs 2–4
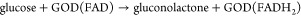
2
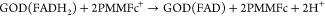
3
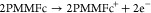
4The glucose is oxidized through immobilized GOD; next, the FAD cofactor donates electrons to the ferrocene moiety from PMMFc that mediates the transport of electrons to the electrodes. Cyclic voltammetry (CV) was conducted to gain preliminary insights into the electrochemical behavior of the PMMFc hydrogel. PLA/Au/PMMFc exhibited a significant shift in the half-wave potential (*E*_1/2_) from −65 to 180 mV compared to PLA/Au/AFc (Figure 5b). The redox potentials of ferrocene derivatives are influenced by the functional groups attached to the ferrocene moiety.^65^ In this study, the positive shift in the redox potential indicates a reduction in the electron-donating substituents on ferrocene, which can be attributed to the transformation from primary to secondary amines of AFc after NHS coupling. This covalently bound ferrocene moiety in the PMMFc hydrogel matrix exhibited negligible current decrease and symmetric redox peaks over 50 continuous CV tests in PBS, demonstrating excellent stability and compatibility with the zwitterionic polymer system (Figure S9). Furthermore, an increase in oxidation and a decrease in reduction current were observed after adding 5 mM glucose to the CV measurement (Figure 5c), indicating the characteristic response of Fc-mediated enzymatically catalyzed oxidation reactions.^66^

The glucose calibration curve was calculated from a +0.24 V chronoamperometric experiment with the PLA/Au/PMMFc-GOD electrode, demonstrating a linear detection range of 2–16 mM with a sensitivity of approximately 5.48 μA cm^–2^ mM^–1^ (Figure 5d). Additionally, the electrode’s continuous monitoring performance was evaluated in the PBS buffer with 5 mM glucose under +0.24 V for over 12 h. The current response maintained stability throughout the continuous measurement, with over 87% of the initial response current maintained (Figure 5e). Moreover, the MN-based biosensor is specifically designed to operate in a complex ISF environment, requiring excellent selectivity against electroactive interferences. To evaluate selectivity, physiological levels of relevant electroactive constituents of human ISF were tested: 200 μM UA, 50 μM AA, and 30 μM AP.^19,67^Figure 5f shows the chronoamperometric response to the successive additions of UA, AA, and AP. The stable response current indicates negligible interference from these substances on the high response observed from 5 mM glucose, confirming the high selectivity of the PMMFc-GOD system, which must benefit from the low potential used in the measurement.^68^

### Antifouling Property of the Zwitterionic Hydrogel Sensing Layer

**Figure 6:**
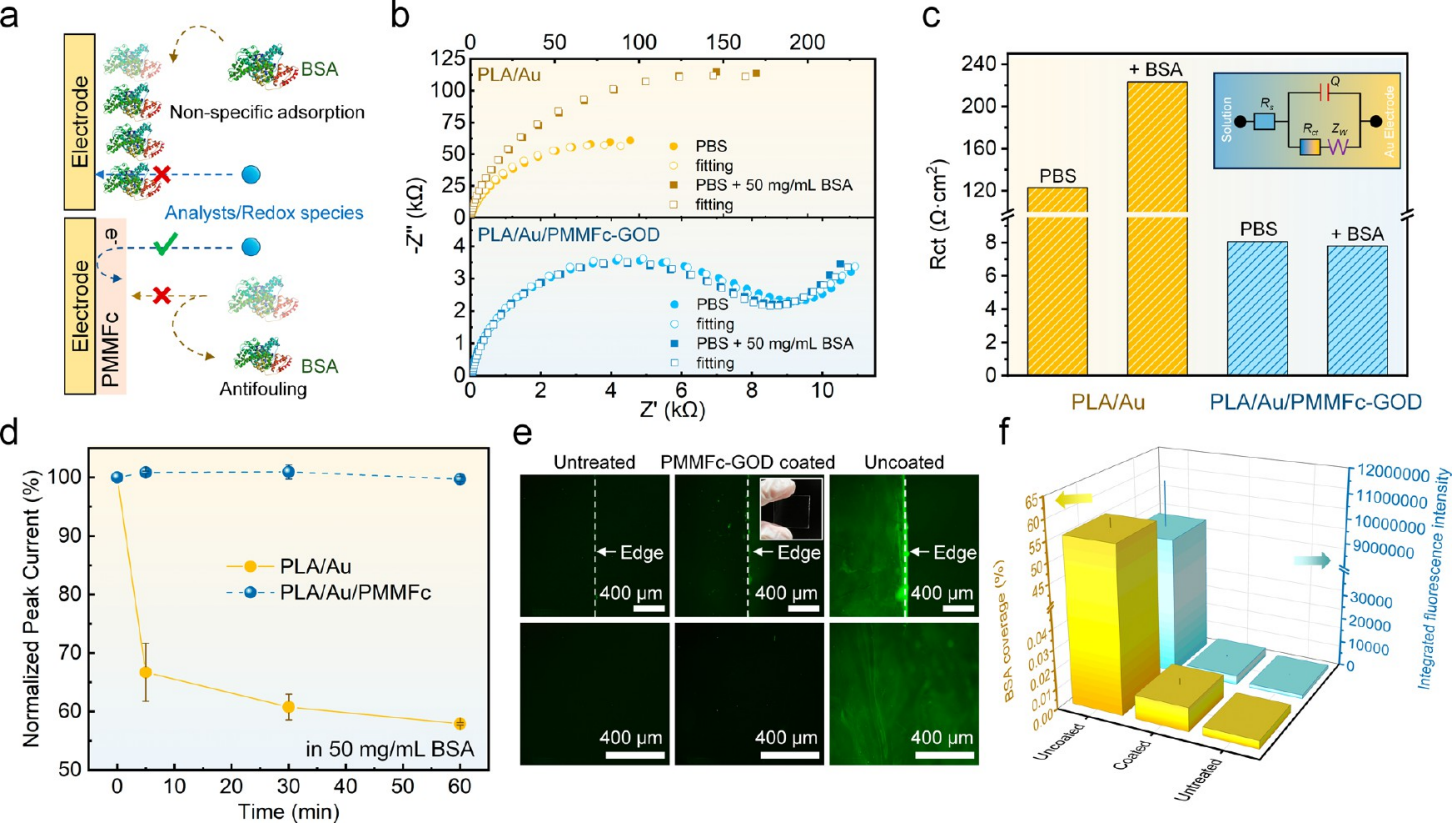
Antifouling behavior evaluation of the PMMFc-GOD hydrogel sensing layer. (a) Schematic of nonspecific adsorption on the electrode. (b) Nyquist curves of bare and zwitterionic hydrogel-coated electrodes before and after the addition of 50 mg mL^–1^ BSA in PBS. (c) Summary of the interfacial transfer resistance from the EIS curve. The inserted model shows the equivalent circuit. (d) Peak current obtained from CV curves of different time points with BSA’s existence. (e) Fluorescence images and (f) the summarized BSA coverage and integrated fluorescence intensity of uncoated, PMMFc-GOD coated, and untreated glass after coincubation with FITC-BSA at 37 °C for 2 h.

Nonspecific adsorption is a common source of signal distortion and offsets in subcutaneous sensors caused by the hydrophobic or electrostatic interaction between biomolecules (e.g., proteins) and electrodes’ surfaces, impeding the diffusion of analytes^55,69^ (Figure 6a). To validate the antibiofouling performance of the PMMFc hydrogel, we initially employed impedance analysis on PLA/Au and PLA/Au/PMMFc-GOD electrodes with BSA as the simulated protein contaminants using EIS (Figure 6b). The impedance data was fitted with an equivalent circuit inserted in Figure 6c using ZSimpWin, and the fitted parameters are summarized in Table S2. The interfacial transfer resistance (*R*_ct_) is crucial, indicating the difficulty of charge transfer at the electrode interface.^70^ The PMMFc-GOD-coated electrode, leveraging the advantageous covalent immobilization of the redox probe, exhibited an *R*_ct_ approximately 1 order of magnitude lower than that of the PLA/Au electrode. In the presence of BSA, the surface of the PLA/Au electrode experienced rapid, nonspecific protein adsorption, leading to an approximate one-fold increase in transfer resistance, which poses challenges for small-molecule substances, such as redox probes or analytes, to reach the electrode surface for electrochemical reactions.^71^ Contrarily, the PLA/Au/PMMFc-GOD electrode demonstrated a nearly constant *R*_ct_ even in complex protein environments.

This antifouling property is also evident in the current evolution from the CV measurements (Figure 6d). The redox current of the bare PLA/Au electrode diminishes to 65% of the initial value within the first 5 min in the presence of simulated protein contaminants (50 mg mL^–1^ BSA). The current continues to decrease over the subsequent hour of exposure. However, the PLA/Au/PMMFc-GOD electrode maintains stability and a high response current for over 60 min, highlighting its superior antifouling properties attributed to the sensing layer’s porous structure and zwitterionic nature. For enhanced intuitive observation of proteins’ adsorption, the PMMFc-GOD hydrogel against nonspecific protein binding was examined visually by the adsorption of fluorescein isothiocyanate-tagged BSA (FITC-BSA) on the bare and hydrogel-coated glass. The uncoated glass was immersed in the PBS buffer containing 50 mg mL^–1^ FITC-BSA for 2 h under fluorescence microscopy, resulting in a bright green color (Figure 6e). Contrarily, the PMMFc-GOD-coated sample exhibited no bright green color, indicating that the zwitterionic interface can resist nonspecific protein in complex biological fluids (Figure 6f).^72^

### Numerical Simulation of Glucose Diffusion within the MN Sensor

**Figure 7:**
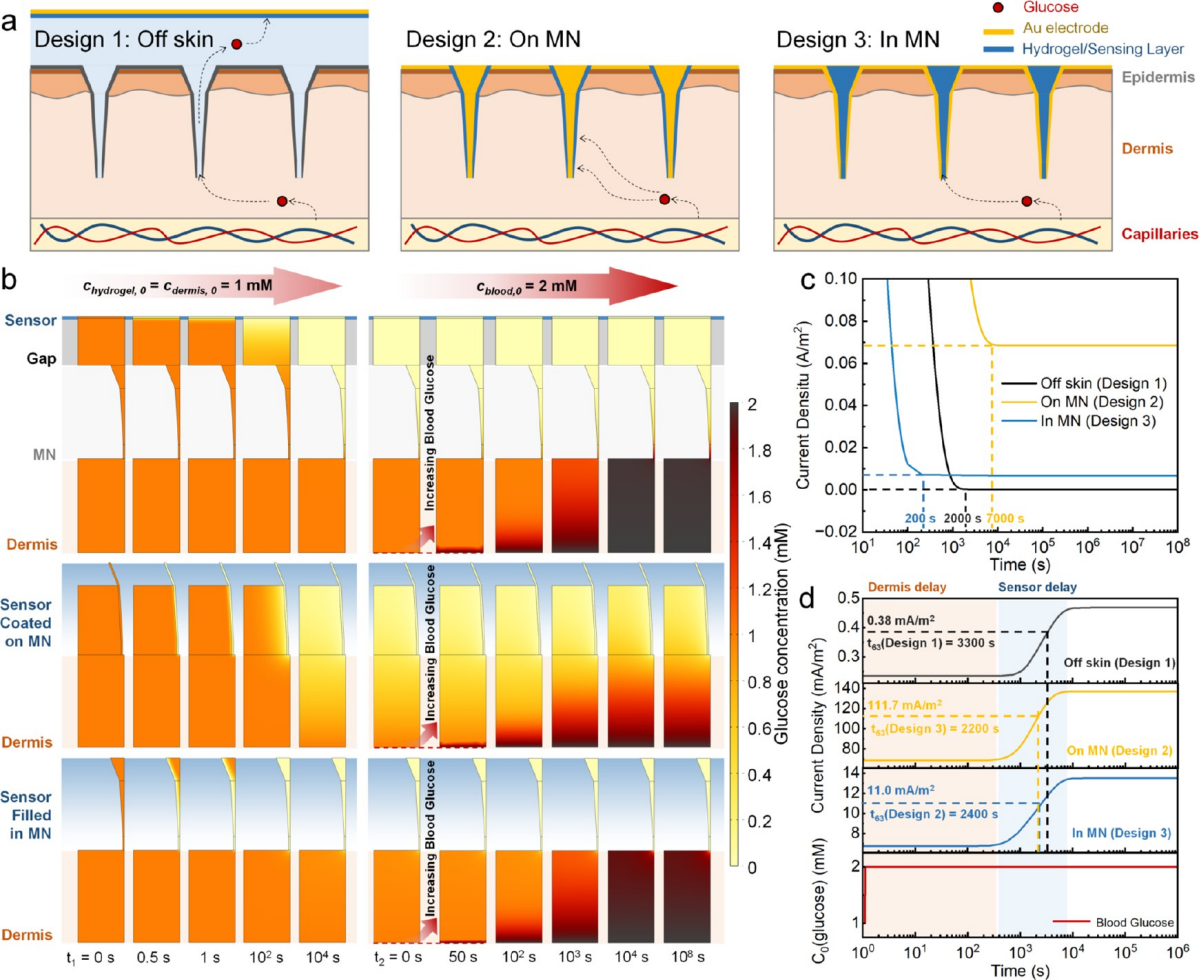
Numerical simulation of glucose diffusion in MN sensors. (a) Schematics of the MN-based transdermal sensor, showcasing various assembly methods of hydrogel sensing layers for glucose sensing within the dermis. (b) 2D snapshots of glucose concentration at different time points. The duration *t*_1_ (time points: 0, 0.5, 1, 10^2^, 10^4^ s) represents the time points of glucose consumption with an initial 1 mM glucose concentration in both hydrogel and dermis layers. The differences between *t*_1_ = 10^4^ s and *t*_1_ = 10^8^ s (not shown) appear to be minimal. The duration *t*_2_ (time points: 0, 50, 10^2^, 10^3^, 10^4^, 10^8^ s) simulates the increase in blood glucose, causing a change in the glucose concentration gradient. (c) Simulated chronoamperometric response within *t*_1_ periods of three different designs. (d) Response delay within the *t*_2_ period when the blood glucose boundary is increased to 2 mM.

The encapsulation of the sensing hydrogel into the hollow MN reduces friction between the sensing layer and subcutaneous tissues, enhances the sensing performance within the 3D electrochemical reaction routes, and eliminates the need for any additional inert protective coating (e.g., chitosan, PVA, Nafion) that may cause a diffusion barrier of the analytes. This core–shell structure has not been reported. Consequently, a preliminary investigation was conducted using numerical simulation to analyze glucose diffusion and electrochemical enzymatic reaction pathways in our proposed design, comparing it with two traditional designs. Three assembly situations of the sensing layer in hollow microneedles were considered: placing it outside the skin (Design 1), situating it on the MN (Design 2), and filling it inside the channel (Design 3) (Figure 7a) without considering inert coatings as barrier layers. These hollow MN models were developed with consistent dimensions, focusing on a two-dimensional axisymmetric cylindrical region near the MNs to minimize the impact of shape-related and neighboring effects on diffusion and sensing behaviors. Detailed structures and simulation parameters are provided in Figure S10a and Table S3.

The glucose concentration initial values of the hydrogel (*c*_hydrogel_) and the sensing layer (*c*_dermis_) were set at 1 mM; at the same time, a +0.24 V potential was applied to the electrode boundaries to simulate clinical chronoamperometric measurement (Figure 7b). Within the initial seconds, glucose is rapidly depleted in the hydrogel, forming a concentration gradient along the height direction and eventually reaching a steady state during the *t*_1_ period. Subsequently, these steady states were set as the initial states, and the blood glucose boundaries (bottom boundary) were assigned to a constant 2 mM concentration to simulate suddenly increasing blood glucose levels. Over *t*_2_ duration, glucose gradually diffused into the upper sensing hydrogel through the dermis, forming a steady-state concentration gradient confirmed in all designs. The global concentration change resulting from the mutation, initiated at the boundary, is also validated by using a normalized 0-to-1 mM concentration transition. The concentration projections along the *z*-axis are shown in Figure S10b. Across the sensor-dermis interfaces in three designs, a concentration decrease of several orders of magnitude was observed, aligning with the simulation results on a hollow MN sensor.^73^ This phenomenon is attributable to the lower diffusivity of glucose in the dermis compared with its high diffusion coefficient in the gap layer or hydrogel. The formation of this concentration gradient is caused by the direct connection between the low diffusive regions (dermis) and the high diffusive regions (hydrogel or gap).

Figure 7c presents the current responses during the *t*_1_ periods, indicating that hydrogel-filled Design 3 demonstrated the fastest numerical establishment of a steady-state concentration gradient. This advantage stems from a more centralized hydrogel-dermis interface and contributes to a faster equilibrium with a smaller gradient compared to Designs 1 and 2. In the simulation of rising blood glucose levels, the response delay is calculated as the time point to reach 63% of the steady-state glucose concentration during the *t*_2_ period^74−76^ (Figure 7d). In scenarios involving changes in blood glucose, diffusion influences response time delays from dermal and sensing layers. When sensors are positioned in the dermis (Designs 2 and 3), their response time is shorter than that of those placed externally on the skin (Design 1).

Previous studies have barely explored the diffusion within microneedle electrochemical sensors, overlooking the disparity between dermal and buffer conditions. Glucose experiences a reduced diffusion efficiency within the dermal layer by at least an order of magnitude, a stark contrast to the swift and shorter diffusion paths in the beaker or artificial dermis (e.g., agarose gel). This discrepancy contributes to a portion of the response delay, as made evident by our simulation results. Furthermore, we postulated a direct contact between the sensing layer and the dermis for a fair comparison in Design 2. However, applying an additional inert protective coating for on-MN design is common in practical applications and may inadvertently create a diffusion barrier. Moreover, the adhesion of biological fouling to these inert coatings during extended use can drastically reduce the analyte concentration by several orders of magnitude.^77^ Contrarily, Design 3 presents a structurally superior solution, addressing this issue by physically separating the sensing layer from the dermis and eliminating the need for an additional coating layer. This approach facilitates more efficient diffusion and potentially enhances the overall sensor performance.

### Evaluation of the PLA/Au/PMMFc-GOD MN Sensor

Numerical simulations have validated the feasibility and revealed the advantages of hydrogel-encapsulated hollow MN electrodes. This core–shell structure can be easily realized by combining the techniques of MN fabrication, Au metallization, and zwitterionic hydrogel synthesis proposed above. The partition of the MN patch can be accomplished and integrated with a simple antisensitization layer deposition step before activation without requiring additional etching or expensive masks. The sensor preparation can be accomplished by decorating the reference electrode and injecting PMMFc-GOD sol into the hollow channels of working electrodes with extended designs (Figure S11). In this section, a typic 9-MN design (1 RE, 3 CEs, and 5 WEs) is used to study the transdermal diffusion-based glucose sensing behavior and the long-term stability of this MN sensor (Figure S11c). The stability of RE was confirmed for a barely changed open-circuit potential of over 100 000 s (Figure S12).

**Figure 8:**
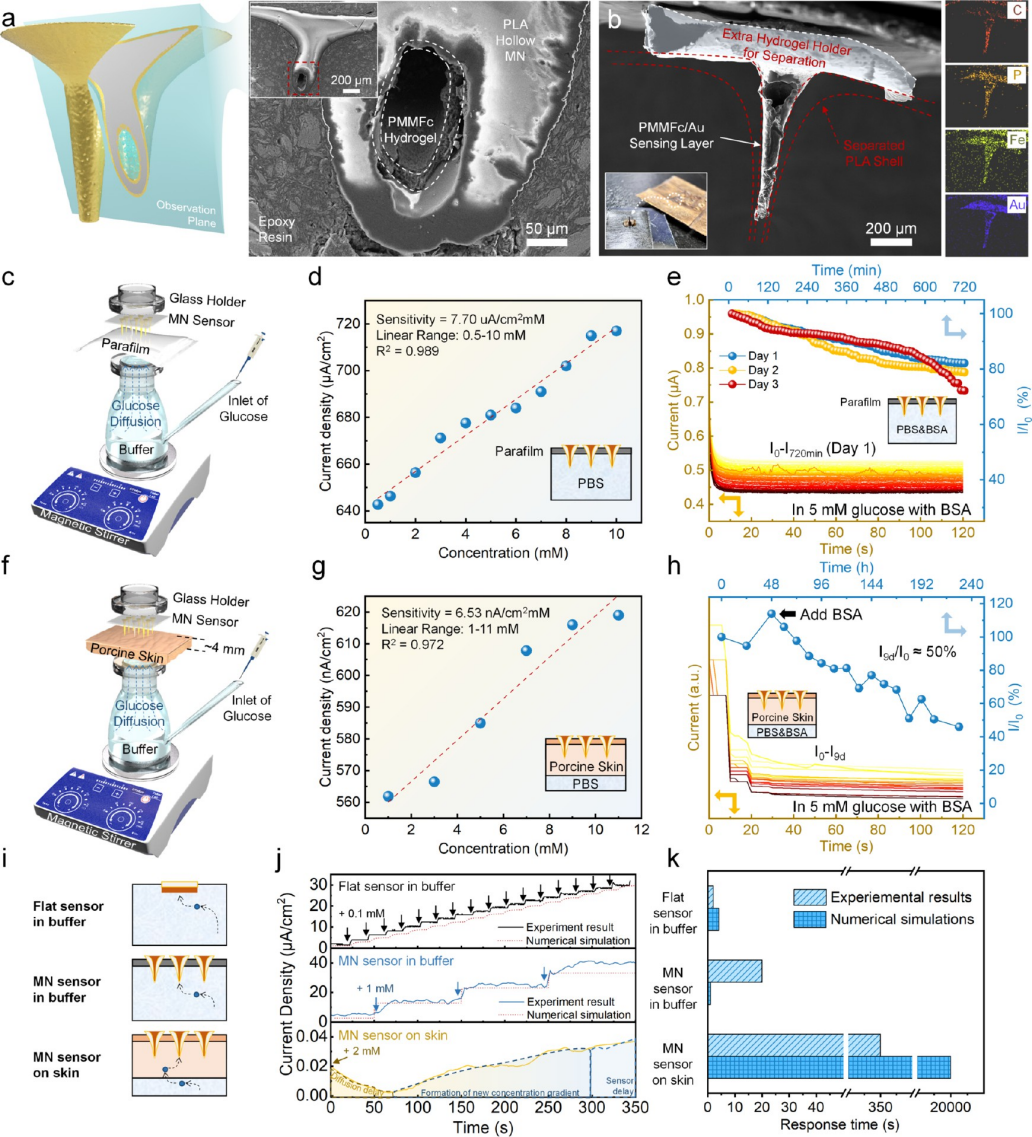
Performance evaluation of the PLA/Au/PMMFc-GOD MN sensor. (a) Cross-sectional observation of hydrogel-encapsulated hollow MN. (b) Separated PMMFc-GOD hydrogel from the channel. (c) Experimental setup and (d) calibration curve derived from chronoamperometric measurement in the buffer. (e) Continuous measurement of the MN sensor in PBS with 5 mg mL^–1^ BSA. (f) Experimental setup and (g) calibration curve derived from chronoamperometric measurement on porcine skin. (h) Long-term stability of MN sensor on porcine skin. (i) Schematics, comparison of the (j) chronoamperometric response behaviors, and (k) response time of three different situations: flat sensor in buffer, MN sensor in buffer, and MN sensor on skin.

Owing to the PLA/Au MNs’ surface was pretreated with cysteamine before WE decoration, the hydrogel could spontaneously fill inside these hydrophilic channels and tightly adhere to the Au electrodes. PMMFc-GOD hydrogel encapsulation in the MN is confirmed using cross-sectional SEM observation and EDS analysis, with a dry thickness of approximately 10 μm (Figure 8a). The robust HMW PLA shell and ample channel space ensure that the hydrogel remains securely anchored within the channel postswelling, without compromising the structural integrity of the external MN. The additional hydrogel was applied to the back of an unannealed WE to verify the Au deposition on the hollow channels for easier separation. Freestanding PMMFc hydrogel with an Au coating peeled with tweezers was observed and analyzed under SEM and EDS. The uniform element distribution confirmed the uniform Au coating and its tight adhesion with the hydrogel layer (Figures 8b and S13).

The MN sensor was initially evaluated by inserting it into a Franz diffusion cell with a stacked parafilm to mimic the epidermis (Figure 8c). The sampling port of the cell was utilized as the glucose inlet, adjusting the glucose level and simulating the diffusion of analytes from the vascular layer at the dermis’ base. A chronoamperometric experiment was subsequently conducted. The sensitivity achieved in the MN sensor was 7.70 μA cm^–2^ mM^–1^ (Figure 8d), comparable to that obtained with planar electrodes. Continuous monitoring was performed in PBS buffer with 5 mM glucose and 5 mg mL^–1^ BSA as protein contaminants. Owing to the superior antifouling property of the zwitterionic hydrogel, the sensors could maintain a stable current response of approximately 80% of every 12 h initial value for over 3 days (Figure 8e). To simulate transdermal monitoring, we applied the MN sensor to porcine abdominal skin (with a thickness of 4 mm) above the buffer using a similar Franz diffusion cell experimental setup (Figures 8f and S14). The glucose calibration curve exhibited a sensitivity of approximately 6.53 nA cm^–2^ mM^–1^ (Figure 8g). The long-term stability of the MN sensor on porcine skin was confirmed for over 9 days, with BSA applied to the diffusion cell from the second day, and the buffer was refreshed every 24 h (Figure 8h).

Owing to the zwitterionic nature’s ability to effectively eliminate the effect of nonspecific adsorption, investigating and comparing the passive-diffusion-dominated electrochemical sensing behavior in three experimental scenarios is essential: a flat sensor in buffer, an MN sensor in buffer, and an MN sensor on the skin (Figures 8i and S15a). Both the flat and MN sensors in the buffer condition demonstrated similar response times at the second level, as confirmed in both experimental results and numerical simulations, which is attributable to the high glucose diffusivity of the PMMFc-GOD hydrogel sensing layer and the short diffusion route in the buffer condition (Figure 8j). According to the simulation results, a reduced response time of the MN sensor is attributable to the concentrated position of the hydrogel in the center of the MN channel, which forms a microelectrode structure. Additionally, the MN sensor necessitates a response time of approximately 20 s in buffer conditions caused by manual fabrication deviations.

Conversely, the numerical simulation in an MN-on-skin model shows a response delay of over 20 000 s, requiring 2000 s even when *D*_dermis_ parametric sweeps from 2 × 10^–6^ cm^2^ s^–1^ (*D*_dermis_(0)) to a value 10 times higher (2 × 10^–5^ cm^2^ s^–1^) (Figure S15b). Despite a decrease in response current and sensitivity caused by the diffusion barrier functions of the dermis layer, the sensor achieved a stable current within a few minutes during the on-skin in vitro test, comparable to an in vitro glucose diffusion model using a tape-stripped porcine skin.^75^ The insertion of the MN can contribute to reducing the response delay and diffusion path caused by the stratum corneum. Furthermore, this rapid response can be attributed to the enhanced permeability of the MN structure, achieved using thicker porcine skin. Based on the four-pathway theory of transdermal penetration, pore and shunt modes of solute diffusion contribute most to the flux.^78^ In our model, the diffusion is simplified to a constant diffusivity; however, the skin extrusion or increased porosity caused by MN insertion or the axial force generated by agitation during testing were not considered, which may cause a mismatch between the experiment and simulation. However, this MN-on-skin diffusion model based on the Franz diffusion cell is a suitable general model and experimental setup for studying the sensing behavior and response time. The experimental results presented above highlight the advantages and limitations of thoroughly evaluating transdermal sensors’ behavior through these setups and are crucial in developing MN-based biosensors and investigating the structural influence on MN sensors’ performance.

### Biosafety and In Vivo Validation of the PLA/Au/PMMFc-GOD MN Sensor

**Figure 9:**
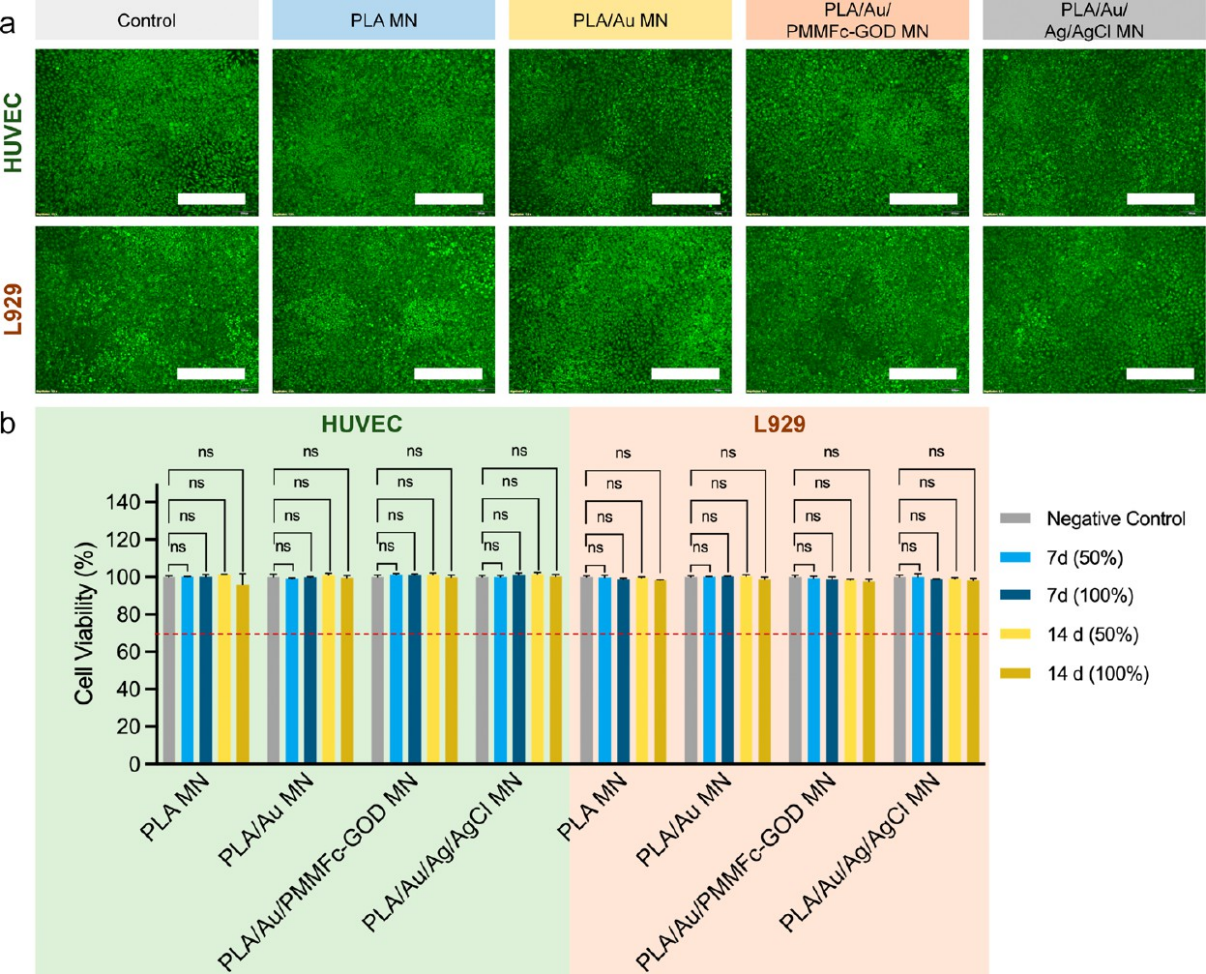
Biocompatibility and cytotoxicity evaluation of MN electrodes. (a) Live/Dead assay results for HUVEC and L929 cells cocultured with different MN electrodes for 7 days. Scale bars represent 500 μm. (b) Cell viability of HUVEC and L929 cells after different incubation periods with extracts (100 and 50% dilution series) of the MN electrodes. The samples include Control, PLA, PLA/Au, PLA/Au/PMMFc-GOD, and PLA/Au/Ag/AgCl MN.

Prior to the in vivo evaluation, the biocompatibility and cytotoxicity of MN electrodes were examined. After coincubating PLA, PLA/Au, PLA/Au/PMMFc-GOD, and PLA/Au/Ag/AgCl MN electrodes with human umbilical vein endothelial cells (HUVEC) and L929 mouse fibroblasts cells for 7 days, Live/Dead stained cells exhibited no significant difference compared to the control group, suggesting excellent biocompatibility of the MN electrodes (Figures 9a and S16). The cytotoxicity evaluation of the electrode extracts using CCK-8 assay was consistent with these results, exhibiting nontoxic effects (Figure 9b), with cell viability well above the acceptable range (>70%) according to ISO 10993-5 and previous literature.^79−81^ The excellent biocompatibility of the MN sensor can be attributed to the biosafety of the chosen materials and fabrication procedures.

**Figure 10:**
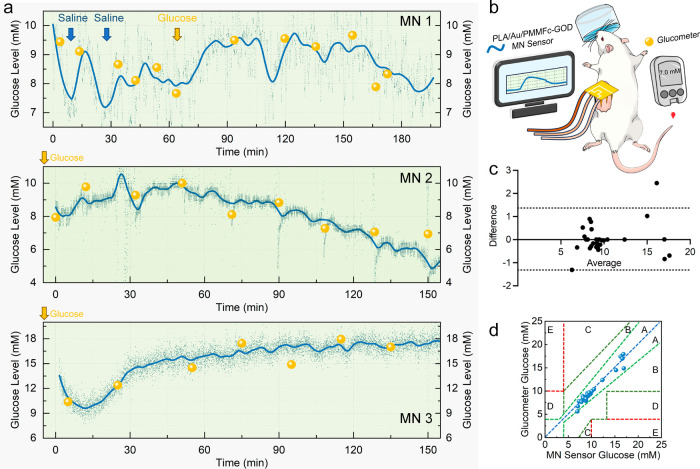
In vivo glucose monitoring performance of PLA/Au/PMMFc-GOD MN sensors. (a) Continuous dynamic monitoring of glucose levels using MN sensors (blue) compared to a commercial glucometer (yellow). (b) Schematic of the experimental setup for glucose monitoring using both sensors. (c) Bland–Altman analysis of the measurement differences between the MN sensor and the glucometer, plotted as a function of the mean of the values. The dashed lines represent the 95% confidence limits. (d) Clarke error grid analysis showing the detection accuracy of glucose measured by the MN sensor compared to the glucometer.

Finally, PLA/Au/PMMFc-GOD MN sensors were tested for in vivo real-time monitoring of glucose dynamics in the ISF of the skin in anesthetized live rats (*n* = 3). During the measurements, blood samples were collected at several time points from the tail vein of each rat, and plasma glucose levels were determined using a commercial glucometer (Medisafe Fit Smile, Terumo, Japan). The results were compared to the data from the MN sensor for a quantitative evaluation. After insertion into the dermis in the lateral abdominal skin of the rats, the MN sensor was allowed to stabilize for 1 h to ensure complete ISF wetting and a stable electrochemical signal. The current of the MN sensors was recorded using chronoamperometry, and two-point calibration was performed prior to measurement (Figures 10 and S18). As shown in Figure 10a, all three MN sensors successfully detected glucose levels in rats and tracked glucose fluctuations with a similar profile. Following stabilization, glucose concentrations were detected to rise rapidly within minutes to half an hour after glucose injection, demonstrating trends similar to those observed with the commercial glucometer and exhibiting good correlation. Although the skin and plasma glucose concentration dynamics varied among the rats, the glucose levels measured by the MN sensor and the glucometer were similar. This can be explained by the paracellular diffusion of the glucose, a small hydrophilic molecule, across the blood-ISF interface,^3^ resulting in almost identical glucose concentrations in both fluids.^4^ Compared to the glucometer, ISF glucose levels detected by the MN sensor usually exhibit a delay of 5–15 min due to the low capillary density of the dermis layer and the diffusion-limited protective layer of traditional dermal sensors.^4^ However, we observed a rapid response, which is possibly attributed to the structural advantages of the hydrogel-filled hollow MN design.

To further assess the accuracy and reliability of the MN sensor, we performed statistical analyses using the mean absolute relative difference (MARD) and Bland–Altman methods. MARD is one of the most commonly used indices for measuring the average difference between a device measurement and the reference measurement for a glucose monitor. The MARD, which was calculated to be approximately 5%, indicated an acceptable deviation from the glucometer readings, validating the sensing ability of the MN sensor.^82^ The Bland–Altman analysis proved the agreement between the two measurement methods, with most of the data points falling within a 95% confidence interval range, indicating little systematic variation between the results obtained with the commercial glucometer and MN sensors (Figure 10c). Finally, Clarke error grid analysis showed that all data points were located in zone A (representing no significant effect on clinical action) and zone B (small altered clinical action on clinical outcome; Figure 10d). These statistical analyses demonstrate that the MN sensor not only correlates well with blood glucose levels measured by the commercial glucometer but also maintains a high level of accuracy and consistency, validating its potential for practical applications in glucose monitoring.

## Conclusions

This study introduces a core–shell MN structure for transdermal electrochemical biosensors, utilizing a hollow inner channel to encapsulate the zwitterionic hydrogel sensing layer for continuous monitoring in the ISF. This design physically isolates the sensing layer from subcutaneous tissue, reducing friction and the need for an additional protective layer, providing an alternative approach for sensor construction and sensing layer protection. The comprehensive fabrication techniques and bottom-up sensor integration were proposed and successfully combined to achieve this structure. Initially, 1000 μm length biocompatible PLA hollow microneedle array patches with high aspect ratios and robust mechanical properties were fabricated through drawing lithography and solution casting. This hollow structure can be metalized with approximately 200 nm thick Au by employing a cyanide-free electroless plating method without intermediate layers. For the sensing layer, we synthesized an MPC-based hydrogel with covalent immobilization capabilities for redox mediators and enzymes coupled with high stability and diffusivity. This hydrogel can be seamlessly filled into the hollow channel and securely adhered to the electrode through a facile drop-casting process, successfully constructing a contaminant-free sensing layer. Numerical simulations of glucose diffusion and electrochemical enzymatic reactions highlight the second-level rapid response characteristics of this MN structure. Moreover, this MN sensor exhibited a 350 s rapid response to glucose in an in vitro evaluation on a 4 mm thick porcine skin and excellent stability in preserving ca. 50% of the initial response after 9 days with protein contaminants. Biosafety evaluations of the MN electrodes demonstrated excellent biocompatibility and negligible cytotoxicity in HUVEC and L929 cells. In vivo experiments further validated the feasibility and effectiveness of the MN sensors, demonstrating accurate and rapid glucose monitoring in live rats. Future work will focus on optimizing the fabrication of fully integrated wireless signal transduction and transmission systems, as well as multiplexed ISF biomarker monitoring, to further enhance the capabilities and applications of wearable MN biosensors.

## Methods

### Materials

Medical grade PLA (*M*_W_ ≈ 220 000) was obtained from Musashino Chemical Laboratory, Ltd. SU-8 3050 was obtained from Kayaku Advanced Materials, Inc. MPC was obtained from the NOF Co., Ltd. Methacrylic acid N-hydroxysuccinimide ester (MNHS), cysteamine, bovine serum albumin (BSA), and FITC-BSA were obtained from Sigma-Aldrich. Sodium hydroxide (NaOH), hydrogen tetrachloroaurate tetrahydrate (HAuCl_4_·4H_2_O), hydrogen hexachloroplatinate hexahydrate (H_2_PtCl_6_·6H_2_O), sodium tetrahydroborate (NaBH_4_), hydrogen peroxide (H_2_O_2_), potassium hexacyanoferrate trihydrate (K_4_Fe(CN)_6_·3H_2_O), potassium hexacyanoferrate (K_3_Fe(CN)_6_), uric acid (UA), L(+)-ascorbic acid (AA), p-acetaminophen (AP), chloroform (CHCl_3_), α,α′-azobisisobutyronitrile (AIBN), amino ferrocene (AFc), sodium hydrogen carbonate (NaHCO_3_), phosphate-buffered saline (PBS, pH 7), 1,4-butanediol diglycidyl ether (BDDE), D-(+)-Glucose, Dulbecco’s modified Eagle’ medium (DMEM), and fetal bovine serum (FBS) were purchased from Wako Pure Chemical Industries, Ltd. Trimethylstearylammonium chloride and polyvinylpyrrolidone (PVP) were purchased from Tokyo Chemical Industry Co., Ltd. Glucose oxidase (GOD, >100 U mg^–1^, EC 1.1.3.4) from *Aspergillus niger* was obtained from MP Biomedicals, Inc. Ag/AgCl ink (cat. no. 011464) was purchase from BAS (Japan). Penicillin–Streptomycin (10 000 U/mL) (P/S) was purchased from Thermo Fisher Scientific. HUVEC was purchased from Lonza (USA). L929 cells were purchased from the Riken Cell Bank (Japan). All of the reagents and solvents were used without further modification or purification. The artificial ISF was prepared according to an early report.^83^

Fresh porcine skin was obtained from a local slaughterhouse (Tokyo Shibaura Zouki Co., Tokyo, Japan). The fat tissue was trimmed by using a dermatome and stored at −30 °C until it was needed. The porcine skin was shaved and equilibrated in PBS prior to use.

### Fabrication of the MN-Based Sensor

#### Preparation of the PLA Hollow Microneedles

Drawing lithography (DL) and solution casting methods were employed to fabricate the PLA hollow MN array, as shown in Figure 2a. First, silicon (Si) pillars were fabricated using a Dicing Saw DAD-522 (DISCO Inc.). These pillars were assembled and combined on a silicon wafer substrate to create the desired microneedle array design. Glass slides were spin-coated with SU-8 3050 at 1000 rpm for 30 s, followed by a soft bake at 120 °C for 15 min to enhance viscosity and processability by evaporating solvents. Subsequently, the SU-8-coated substrate was transferred onto a preheated substrate at 58 °C. An array of Si pillars of various sizes (300, 500, and 1000 μm), drawing at different speeds (25 or 50 μm s^–1^), can be utilized to fabricate an array of SU-8 MNs with good consistence (Figure S2). The typical parameters for drawing lithography using a Si pillar involve lifting up 300 μm pillars at a speed of 50 μm s^–1^ to form the SU-8 MN structures. The drawing process was stopped after the desired height was achieved, and the *Z*-axis position of the pillars was maintained for 30 min, separated at a drawing rate of 1000 μm s^–1^, resulting in arrays of SU-8 MNs. Following the separation, the SU-8 MNs were exposed to 365 nm UV light for 20 min and annealed at low temperatures as a simplified hard-bake procedure to enhance the strength of the microneedle structures. Subsequently, a prepared PLA/CHCl_3_ solution (7.5 wt %) was drop-cast onto the SU-8 MNs. As CHCl_3_ completely evaporated, the PLA coating was gently peeled from the SU-8 substrate using tweezers, resulting in a stand-alone PLA hollow MN patch. The diameter and surface roughness of the PLA hollow MN can be fine-tuned by adjusting the evaporation rate through temperature control (Figure S3). Typically, the PLA hollow MN with a sharp tip and smooth surface can be achieved at room temperature (RT). However, using this method, the SU-8 MN mold must have a height of more than 3 mm to create the hollow PLA structure.

#### Realization of Au Metallization

To enable the PLA hollow MN to function as an electrode and to load the sensing layer, a cyanide-free Au electroless plating process was employed to achieve uniform metallization. The PLA MN was chemically etched using a 1 M NaOH solution at 50 °C for 5 min to increase the surface area and hydrophilicity prior to electroless plating. The plating process consists of sequence activation, sensitization, and plating steps. First, the PLA substrate is immersed in a 3.2 mM trimethyl stearyl ammonium chloride aqueous solution for 1 min to activate the surface. Subsequently, the substrate is dipped into a Pt colloid solution for 1 min to sensitize the surface. The Pt colloid solution is prepared by mixing 0.05 mM H_2_PtCl_6_·6H_2_O, 2 mg mL^–1^ PVP, and 2 μM NaBH_4_ aquas solutions. After activation and sensitization, the PLA substrate is subjected to ultrasonication and rinsed with deionized water for 1 min. Finally, the substrate is immersed in the Au electroless plating solution containing 3.36 mg mL^–1^ HAuCl_4_ and 0.2% H_2_O_2_ for 5–20 min at 0 °C, RT, and 50 °C under agitation. Notably, within a few minutes, bright, shiny, and smooth Au coatings form on the PLA surface (Figure S4). Following the plating process, the PLA/Au substrate is washed and annealed at 150 °C for 1 h to enhance the connection between the PLA and Au. The resulting PLA/Au structure can be stored under ambient conditions for later use. For the circuitization and functional partitioning of the PLA MN patch, the oil ink is deposited on the PLA MNs patch to prevent the attachment of the Pt colloid to the PLA substrate and then conduct the same activation and sensitization steps. Before the plating, the PLA MN patch was allowed to be ultrasonicated in ethanol for 5 s to remove the oil ink.

#### Fabrication and Optimization of the Zwitterionic-Based Redox Polymer Hydrogel

Poly(MPC-*co*-MNHS) (PMS) copolymer was synthesized *via* free radical polymerization, following our previously reported method.^40^ A mixture of 0.5 M MPC, 0.5 M MNHS monomers, and 0.005 M AIBN as the initiator was polymerized in 6 mL of CHCl_3_ at 59 °C for 24 h under an argon atmosphere. The resulting white precipitates were collected, washed with CHCl_3_, filtered, and vacuum-dried. The obtained PMS powders were stored at −30 °C until further use.

To synthesize the PMMFc copolymer, 50 mM AFc and 26 mg mL^–1^ PMS (0.1 M NaHCO_3_ solution, pH 8.5) were reacted under continuous stirring at 37 °C for 5 days. Subsequently, the PMMFc colloid was dialyzed in DI water for 2 days and freeze-dried, yielding reddish-brown powders for subsequent applications.

#### Fabrication of the Electrode Using the PMMFc Redox Polymer Hydrogel

The PLA/Au electrodes were cleaned by performing cyclic voltammetry (CV) in 0.5 M H_2_SO_4_, scanning from −0.3 to 1.6 V until a stable response was achieved. Subsequently, the PLA/Au electrodes were immersed in a 20 mM cysteamine aqueous solution to enable the self-assembly of amine groups on the Au surface. The PMMFc powders were dissolved in a 0.1 M NaHCO_3_ aqueous solution at 170 mg mL^–1^ concentration to prepare the enzyme-containing redox hydrogel. Subsequently, 50 mg mL^–1^ of GOD and 20 wt % of BDDE were added to the PMMFc solution. The PMMFc-GOD solution was stirred continuously for 24 h at 4 °C. The resulting sol was drop-cast onto the aminated PLA/Au electrodes and dried in a fume hood for 24 h. The hydrogel was applied at a density of 50 μL cm^–2^. In the case of MN electrodes, each hollow channel received an injection of 0.1 μL sol from the base side. For the reference electrode, the PLA/Au MN electrodes are dip-coated with Ag/AgCl ink, followed by drying at 120 °C for 20 min. Before electrochemical measurements, the electrodes were soaked in PBS to ensure the equilibrium swelling of the hydrogel.

### Characterization

#### Geometry Measurements

The SU-8 MN array, PLA hollow MNs, and PLA/Au electrodes were observed by using SEM to visualize their structures. For cross-sectional observation, the samples were embedded in EP-200 epoxy putty (RectorSeal Co.) or ET epoxy resin (ITW Performance Polymers). The samples were sequentially polished using progressively finer grades of sandpaper (400, 1000, 2000, and 5000#) and finally polished with 0.1 μm diamond and 0.05 μm alumina paste. Prior to SEM observations, samples were sputter-coated with Pt. The length, diameter, tip angle, bottom width of the PLA hollow MNs, and thickness of the Au layers were measured using ImageJ.

#### Mechanical Evaluation and Porcine Insertion Test

The mechanical properties of MNs were evaluated by using an FSA 0.5KE 50N motorized test stand (Imada Inc.) at a compression rate of 50 μm s^–1^. The in vitro insertion test was performed on porcine debridement back skin. The skin was harvested and frozen before testing. The defrosted tissue was stored in PBS to prevent drying. A manual force was applied to the porcine skin for 1 min, and the resulting pores were observed after removal of the MNs. The mechanical stability of the PLA/Au MN electrodes was assessed in terms of their conductivity and morphological integrity, particularly against subcutaneous friction. This assessment involved applying the same electrodes to the porcine skin 50 times, with each application lasting 1 min. The conductivity and surface morphology of the electrodes were examined both before and after application through a four-probe resistance meter (Loresta-GXII MCP-T710, Nittoseiko Analytech) and SEM, respectively.

#### Long-Term Immersion Test

The degradation and stability of the PLA/Au electrodes were assessed through a long-term immersion test in artificial ISF. The PLA and PLA/Au samples were immersed in 2.5 mL of artificial ISF per 1 cm^2^ exposed surface area and maintained at 37 °C for 66 days. The electrodes were dried and weighed at the end of each day during the first 3 weeks and at the end of the fourth, fifth, seventh, and ninth weeks. The conductivity of the PLA/Au electrodes was measured at the fixed test points using an electric meter after each drying process.

#### Characterization of Physical and Chemical States of PMS and PMMFc

To observe the surface structure, 10 mg mL^–1^ of the PMMFc hydrogel was drop-cast onto a 5 × 5 mm^2^ silicon wafer and then dried under vacuum. The resulting surface was observed by using SEM without requiring an additional conductive layer. The size distribution of the microgel particles in the 10 mg mL^–1^ PMMFc solution was analyzed using dynamic light scattering (DLS). For the components analysis, PMS copolymers were hydrolyzed in 0.1 NaHCO_3_ for 5 days and dialysis in DI water to obtain the water-soluble polymer. However, PMMFc cannot be dissolved in common solvents and instead forms sols or suspensions. Therefore, the elemental composition and chemical states of PMMFc were characterized by using energy-dispersive X-ray spectroscopy (EDS) and Fourier transform infrared (FT-IR) spectroscopy instead of ^1^H NMR.

#### Swelling Behavior of the PMMFc Hydrogel

The swelling behavior of the PMMFc hydrogel was investigated by measuring the weight change of the hydrogel in both DI water and PBS buffer. The swelling ratio was calculated using eq 5
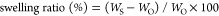
5where *W*_S_ represents the weight of hydrogels after swelling for different periods, and *W*_O_ represents the weight of dried hydrogels. For the swelling–deswelling cycles of the PMMFc hydrogel, after measuring the swelling ratio, the PMMFc hydrogel was stored in a fume hood for 24 h to evaporate the water. After measuring the weight of the deswelled PMMFc hydrogel, the hydrogel was allowed to be swelled again in DI or PBS for 24 h. This swelling–deswelling cycle was repeated for 2 w.

#### Glucose Diffusion Coefficient through the PMMFc Hydrogel

The diffusion coefficient of glucose through the hydrogel matrix was assessed by using a side-by-side Franz diffusion chamber setup. A thin layer of the PMMFc hydrogel (0.3 mm thickness, 15 mm diameter) separated the donor and receptor chambers. In the donor cell, a solution containing 1 mM glucose in PBS was introduced; on the other hand, PBS was added to the receptor cell. Samples were collected from both chambers at various time intervals, and the glucose concentration was determined using an assay kit from Dojindo Laboratories Co., Ltd. To calculate the diffusion coefficients, Fick’s second law was employed based on the change in glucose concentration over time, as described by eq 6
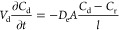
6where *l* is the thickness, *A* is the area of the membrane, *D*_e_ is the effective diffusion coefficient of the hydrogel, and *V*_d_ is the volume of the donor phase. With the use of eq 6, the glucose diffusion coefficients were calculated by measuring the concentration of glucose in both the donor and receptor phases at various times.

For the simulation on verification of glucose diffusion through the PMMFc hydrogel membrane, a two-dimensional model with a 0.3 mm thick hydrogel membrane located in the middle of the channel between two cells was constructed using COMSOL Multiphysics. The donor cell region’s initial glucose concentration was 1 mM. The glucose concentrations in the receptor cell and the hydrogel membrane were set to 0 mM. The average glucose concentration in the receptor was recorded from 0–3600 s with an interval time of 100 s.

### Electrochemical Measurements

Electrochemical measurements were conducted at room temperature using a multichannel potentiostat MultiPalmSens4 for cyclic voltammetry (CV) and chronoamperometry. Electrochemical impedance spectroscopy (EIS) measurements were performed with a Princeton Applied Research VersaSTAT 4 potentiostat. Working electrodes included Au rods (5 mm, ALS Co., Ltd.), PLA/Au, or PLA/Au/PMMFc-GOD samples, with a platinum wire serving as the counter electrode and Ag/AgCl (3 M NaCl) as the reference electrode.

#### Electrochemical Behavior of PLA/Au and PLA/Au/PMMFc Electrodes

Before measurements, the Au rod and PLA/Au electrodes were washed using CV scanning in 0.5 M H_2_SO_4_ from −0.3 to 1.6 V until a stable response was obtained. An additional CV cycle from 0 to 1.6 V was recorded after cleaning. Subsequently, both types of electrodes underwent CV in PBS containing 10 mM Fe(CN)_6_^3–/4–^ at different scanning rates ranging from 20 to 100 mV s^–1^. To compare the redox potential of AFc and PMMFc, the hydrogel-coated electrode was subjected to CV at 100 mV s^–1^ in PBS and compared to the CV of the PLA/Au electrode in PBS containing 5 mM AFc. The stability of the PMMFc hydrogel was assessed on PLA/Au electrodes through CV scanning from 0 to 0.4 V for 50 cycles, with the last 48 cycles recorded.

#### Glucose Sensing Behavior of PLA/Au/PMMFc-GOD Electrodes

The glucose oxidation behavior of the PLA/Au/PMMFc-GOD electrodes was investigated using CV scanning from −0.2 to 0.6 V at a scan rate of 100 mV s^–1^. CV curves were recorded before and after the addition of 5 mM glucose to PBS. Current versus glucose concentration calibration curves were obtained by chronoamperometry measurements at +0.24 V and titrating glucose into PBS under stirring at 200 rpm. To examine the effects of interferences, sequential additions of 200 μM uric acid, 50 μM ascorbic acid, 30 μM acetaminophen, and 5 mM glucose were added to the buffer solution, with the potentiostat recording the *i*–*t* curve for each addition. The effective active surface area of the PLA/Au electrode can be estimated by calculating the integrated area of the reduction peak during CV scanning in 0.5 M H_2_SO_4_.

#### Antifouling Evaluation

The antifouling property of the electrodes was evaluated by performing CV in a PBS solution with 10 mM Fe(CN)_6_^3–/4–^ as the redox probe, followed by adding 50 mg mL^–1^ BSA to the solution. CV curves were measured after 5, 10, 30, and 60 min, and the oxidation currents were recorded and analyzed. EIS measurements were conducted to assess the effect of protein adsorption on the electrodes. The open-circuit potential (OCP) of the working electrodes was measured in the solution for at least 1800 s, until a potential change of less than 1 mV occurred within 120 s. Subsequently, EIS testing was performed from 10^–2^ to 10^5^ Hz at OCP. The Nyquist curve was fitted using an equivalent circuit and analyzed using ZSimpWin 3.60.

For the fluorescence observation of the protein adsorption, the PMMFc-GOD solution was diluted five times using PBS and spin-coated on the oxygen plasma pretreated glass. The untreated, uncoated, and PMMFc-GOD-coated glasses were incubated in PBS buffer with 5 mg mL^–1^ FITC-BSA at 37 °C and followed by rinsing using DI water and dried before observation. Fluorescence microscopy was used to observe the samples under the same contrast and brightness. The images of the samples were channel-separated and calculated using ImageJ to obtain the protein coverage and fluorescence intensity.

#### Stability Evaluation

The sensing responses for glucose were obtained from the currents at 120 s in a 5 mM glucose solution in PBS, with an applied working potential of +0.24 V versus Ag/AgCl. The PLA/Au/PMMFc-GOD flat sensors were tested every hour for 12 h. The PLA/Au/PMMFc-GOD MN sensors were tested every 10 min for 12 h after penetrating the parafilm-sealed Franz diffusion cell filled with an additional 5 mg mL^–1^ BSA. For the stability evaluation on the transdermal sensing platform, the MN sensors were applied to a 4 mm thick debrided porcine abdominal skin, and the current was recorded every 12 h for 9 days. The *I*/*I*_0_ ratio was calculated to measure the current response’s degradation rate.

### Biosafety and In Vivo Evaluation

#### Biocompatibility and Cytotoxicity Evaluation

The biocompatibility of the microneedle array was evaluated using Live/Dead staining (InvitrogenTM, Thermal Fisher) on HUVEC and L929 cells cultured in a DMEM medium. HUVEC and L929 cells (1 × 10^4^ well^–1^) were seeded on a 6-well plate and cultured for 7 days. During this period, presterilized microneedle patches of PLA, PLA/Au, PLA/Au/PMMFc-GOD, and PLA/Au/Ag/AgCl MN electrodes were immersed and cocultured with the cells for durations of 5 and 7 days at 37 °C. Images were captured using a fluorescence microscope and subsequently analyzed with ImageJ to count the live cells.

The cytotoxicity of the MN patches was examined by preparing sample extracts and quantified using CCK-8 colorimetric assays (Donjido Laboratories; Figure S17). MN electrodes were immersed in DMEM medium with 1% P/S for 7 and 14 days at an exposure area of 50 mm^2^ mL^–1^.^84^ Extracts were then supplemented with 10% FBS. HUVEC and L929 cells (5 × 10^3^ well^–1^) were seeded into a 96-well plate and cultured for 24 h. The medium was then replaced with MN electrode extracts and cultured for an additional 24 h. Following the CCK-8 assay protocol, a plate reader measured the absorbance at 450 nm. Cell viability was calculated relative to that of the control group.

#### In Vivo Glucose Monitoring Using MN Sensor

In vivo glucose testing was conducted by using the PLA/Au/PMMFc-GOD MN sensor connected to an external potentiostat (Autolab PGSTAT302N, Metrohm). The procedure began by shaving the hair on the lateral abdomen of 10-week-old male Wistar rats (weight: 230–270 g). The sensor was adhered to the skin and manually inserted into the dermis using gentle finger pressure. To enhance sensor stability and minimize noise from movement, the microneedle sensor was secured to the rat using medical tape (4578, 3M, Japan). The in vivo dynamic ISF glucose monitoring performance of the PLA/Au/PMMFc-GOD MN sensor was compared with that of a commercial blood glucose meter (Medisafe Fit Smile, Terumo, Japan). To reduce signal/noise fluctuations, the MN sensor system did not record glucose levels for the first hour. The rats’ glucose levels were artificially increased by injecting 1 mL 20% w/v glucose solution.^79^ Following the injection, glucose levels were measured and recorded using both the MN sensor and a commercial glucose meter. Blood samples were collected from the tail vein, and the glucose concentration was calibrated and measured using a commercial glucometer. The MARD is calculated using the following equation^85^
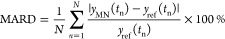
7where *y*_MN_*(t*_n_) is the value measured by the MN sensor, *y*_ref_*(t*_n_) is the value measured by the reference glucometer at different time points, and *t*_n_ are the times when reference measurements are available.

#### Ethics Statement

All animal experiments were conducted following the guidelines and regulations set by the University of Tokyo/Office for Life Science Research Ethics and Safety and were approved by the committee (Approval number: A2024E008). The experiments were designed in accordance with the Japanese Animal Protection and Management Law. Male Wistar rats (230–270 g, 10 weeks of age; SLC Inc., Hamamatsu, Japan) were housed at the University of Tokyo animal facilities and maintained on a 12 h light/12 h dark cycle. Animal handling and reporting adhered to the ARRIVE guidelines.^86^

### Numerical Simulation

Simulations were conducted by using the COMSOL Multiphysics finite-element software package. The transport of diluted species, chemical reactions, and electroanalysis modules were used and coupled under either transient or steady-state conditions. The efficient and time-saving two-dimensional (2D) axisymmetric model was established with an overall mesh density of approximately 100 000 cells.

For the simulation of transdermal diffusion of glucose, the glucose molecules initially diffuse from the dermis toward the enzymatic sensing hydrogel located either outside the skin, on the MN, or inside a hollow MN. First, we define the fundamental physical processes within the dermis and channel-filling hydrogel domains and guarantee flux continuity across the interfaces. The Fick’s second law describes the analyte diffusion
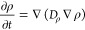
8where ρ represents the analyte concentration (Glucose, Gluconolactone, PMMFc^+^, and PMMFc) with diffusivity *D*_ρ_ within the corresponding domain (*D*_dermis_ for dermis and *D*_MN_ for MN sensor), in the enzyme layer, the diffusion of enzyme derivatives (*E*_R_, *E*_O_, *E*_Glucose_, and *E*_PMMFc_) within hydrogel layer is disregarded due to their considerably larger size, resulting in substantially lower diffusion coefficients compared to the other relevant small molecules.

After capturing glucose using GOD (FAD), electrons are transferred from the glucose molecule through the PMMFc mediators. Therefore, we employed a ping-pong bibi mechanism to represent the PMMFc-mediated enzymatic reaction on the MN, which can be described as follows:
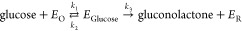
9
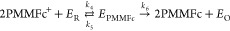
10
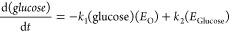
11
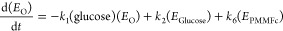
12
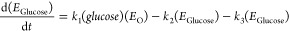
13
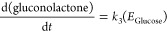
14
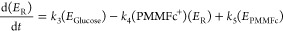
15
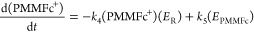
16
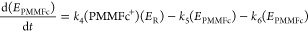
17
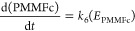
18where *E*_O_ and *E*_R_ are oxidized enzyme GOD (FAD) and reduced enzyme GOD (FADH_2_), *E*_Glucose_ and *E*_PMMFc_ indicate the Michaelis complexes with the bound glucose substrate and PMMFc mediator, and *k*_i_ is the reaction rate constant of the respective reaction, respectively. The reactants (i) GOD (FAD), GOD (FADH_2_), enzyme complexes, and (ii) PMMFc^+^ and PMMFc follow the mass conservation relation as:
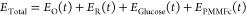
19
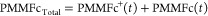
20where *E*_Total_ is the total enzyme concentration. Subsequently, the redox reaction occurs at the electrode surface: the electrons are extracted from PMMFc and collected by the Au electrode.
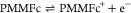
21

The current density for this reaction is given by the electroanalytical Butler–Volmer formalism for an oxidation
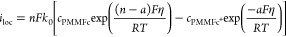
22where *k*_0_ is the standard heterogeneous electron transfer rate, *c*_PMMFc_ is the time-dependent ferrocene concentration at the electrode surface, *F* is the Faraday constant, *R* is the gas constant, *T* is the temperature, α is the transfer coefficient, and η is the overpotential at the working electrode.

## Supporting Information Available

Fabrication of SU-8 MNs arrays using drawing lithography (Figure S1); the SU-8 MNs arrays fabricated under different parameters (Figure S2); PLA hollow microneedles obtained at different preform temperatures (Figure S3); surface observation of electroless plated PLA/Au surface (Figure S4); characterization of PLA/Au electrodes (Figure S5); electrochemical performance of electroless plated PLA/Au electrodes (Figure S6); characterization of PMS and PMMFc (Figure S7); clucose diffusion coefficient measurement of the PMMFc hydrogel (Figure S8); stability evaluation of PMMFc-GOD hydrogel on PLA/Au electrode (Figure S9); numerical simulation on glucose diffusion using COMSOL (Figure S10); fabrication procedures of PLA/Au/PMMFc-GOD sensor (Figure S11); long-term stability of the Ag/AgCl reference electrode (Figure S12); morphologic and elemental characterization of PLA/Au/PMMFc-GOD MN sensor (Figure S13); experimental setups and the chronoamperometric measurement of glucose (Figure S14); experimental setups and numerical simulation on glucose diffusion (Figure S15); biocompatibility evaluation of MN electrodes (Figure S16); schematic diagrams representing the measurement of cytotoxicity of MN electrodes using extraction medium and CCK-8 assay (Figure S17); and in vivo glucose monitoring performance of PLA/Au/PMMFc-GOD MN sensor (Figure S18). A list of band assignments for FT-IR spectra (Table S1); summary of EIS fitting parameters for different samples (Table S2); and parameters used in COMSOL simulation (Table S3) (PDF)
