# Supplementary material for: Biocompatible Core–Shell Microneedle Sensor Filled with Zwitterionic Polymer Hydrogel for Rapid Continuous Transdermal Monitoring
Source: ACS Nano. 2024 Sep 19;18(39):26541–59. doi: 10.1021/acsnano.4c02997 (PMC11447902; doi:10.1021/acsnano.4c02997)
Supplement: Supplementary file 1 — nn4c02997_si_001.pdf [file nn4c02997_si_001.pdf]

Supporting Information for

Biocompatible core-shell microneedle sensor filled with zwitterionic polymer hydrogel for rapid continuous transdermal monitoring

*Shicheng Zhou[1], Yutaro Chino[2], Toshihiro Kasama[1,3], Ryo Miyake[1], Shigenobu Mitsuzawa[4], Yinan Luan[1], Norzahirah Binti Ahmad[5], Hiroshi Hibino[5,6] and Madoka Takai[1]**

1. Department of Bioengineering, The University of Tokyo, Tokyo, 113-8654, Japan
2. Sanyo Chemical Industries, Ltd., Kyoto, 605-0995, Japan
3. Institute of Nano-Life-Systems, Institutes of Innovation for Future Society, Nagoya University, Nagoya, 236-0027, Japan
4. Honda R&D Co., Ltd., Saitama, 351-0193, Japan
5. Division of Glocal Pharmacology, Department of Pharmacology, Graduate School of Medicine, Osaka University, Osaka, 565-0871, Japan
6. AMED-CREST, AMED, Osaka, 565-0871, Japan

* Corresponding Author

Email: takai@bis.t.u-tokyo.ac.jp

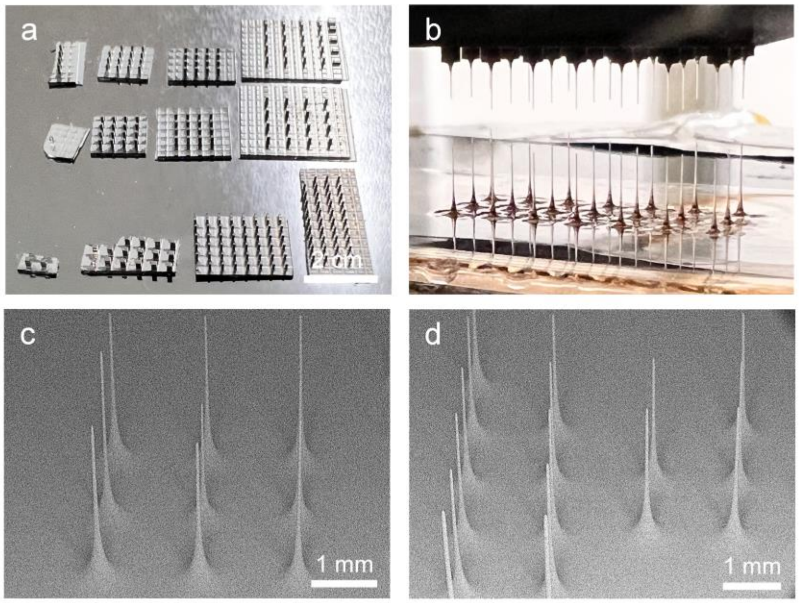

Figure S1. Fabrication of SU-8 MNs arrays using drawing lithography. (a) Si pillars with various designs fabricated using the Dicing Saw. (b) Separated SU-8 MN from Si pillars after solidification. (c) Fabricated 3×3 SU-8 pillar array with 1.6 mm spacing. (d) Examples of scalable pillar arrays of SU-8 MNs with complicated design.

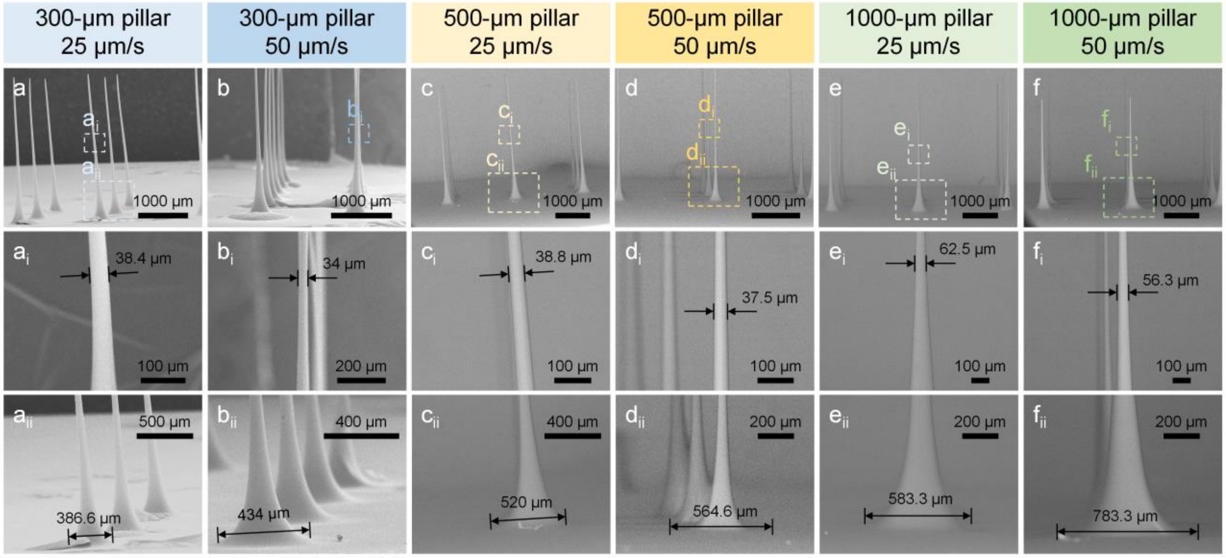

Figure S2. The SU-8 MNs arrays fabricated under different parameters. 300-µm Si pillars lifting at a speed of (a) 25 µm/s and (b) 50 µm/s; 500-µm Si pillars lifting at a speed of (c) 25 µm/s and (d) 50 µm/s; 1000-µm Si pillars lifting at a speed of (e) 25 µm/s and (f) 50 µm/s. Partial enlargements of MNs' middle parts at approximately 1000-µm height areas (a_i-f_i), and bottom parts (a_{ii}-f_{ii}) in (a-f). The temperature is fixed at 58°C while the pillar lifting distance is fixed at 60 mm.

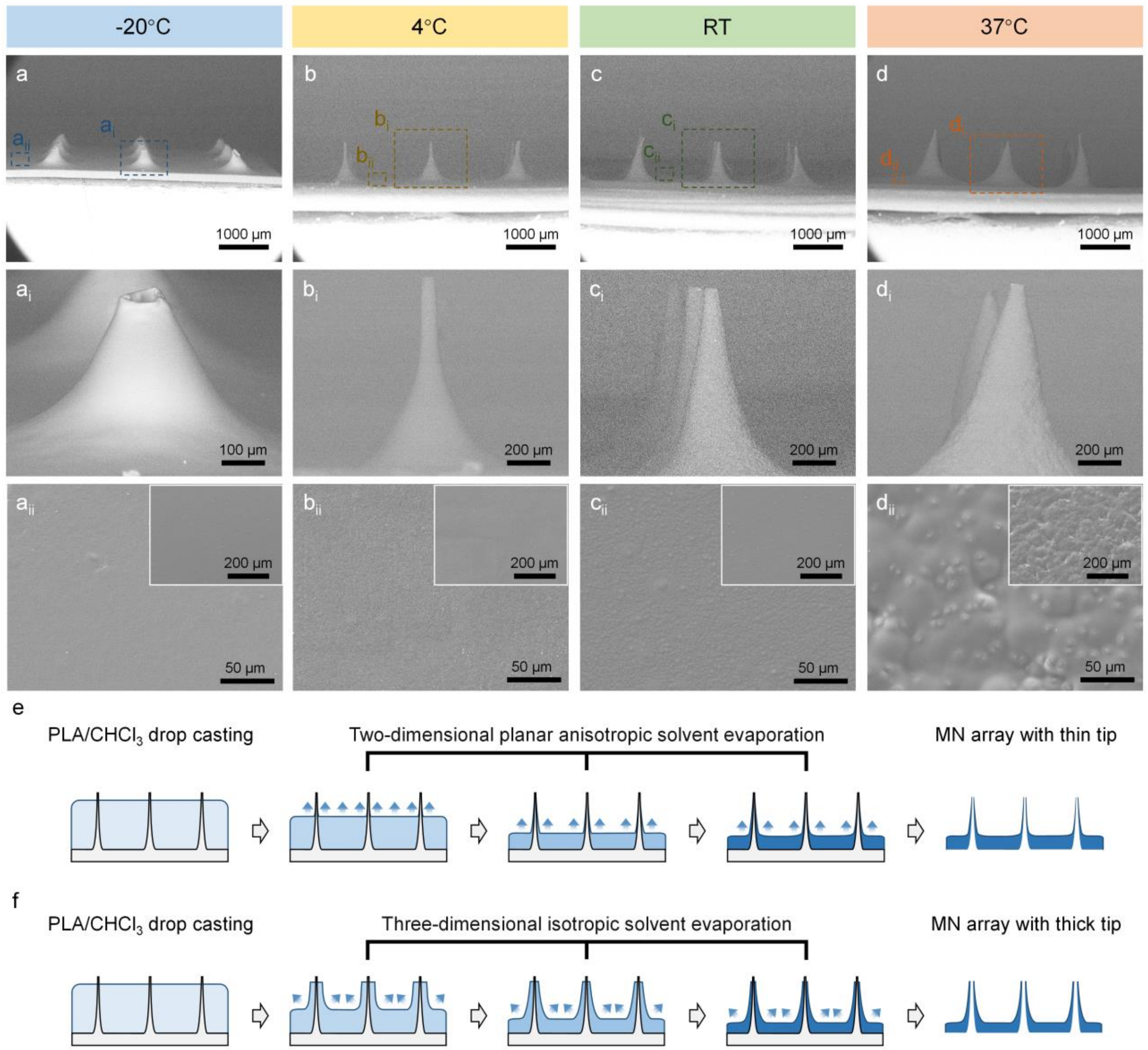

Figure S3. PLA hollow microneedles obtained at different preform temperatures. PLA hollow MNs arrays obtained at (a) -20°C, (b) 4°C, (c) RT, (d) 37°C. (a_i-d_i) Magnified view of single PLA hollow MN from (a-d). (a_{ii}-d_{ii}) surface observation of the flat bottom area from (a-d). The thin tip AN array of PLA formation at relatively low temperature (e) and thick MN array of PLA formation at high temperature (f).

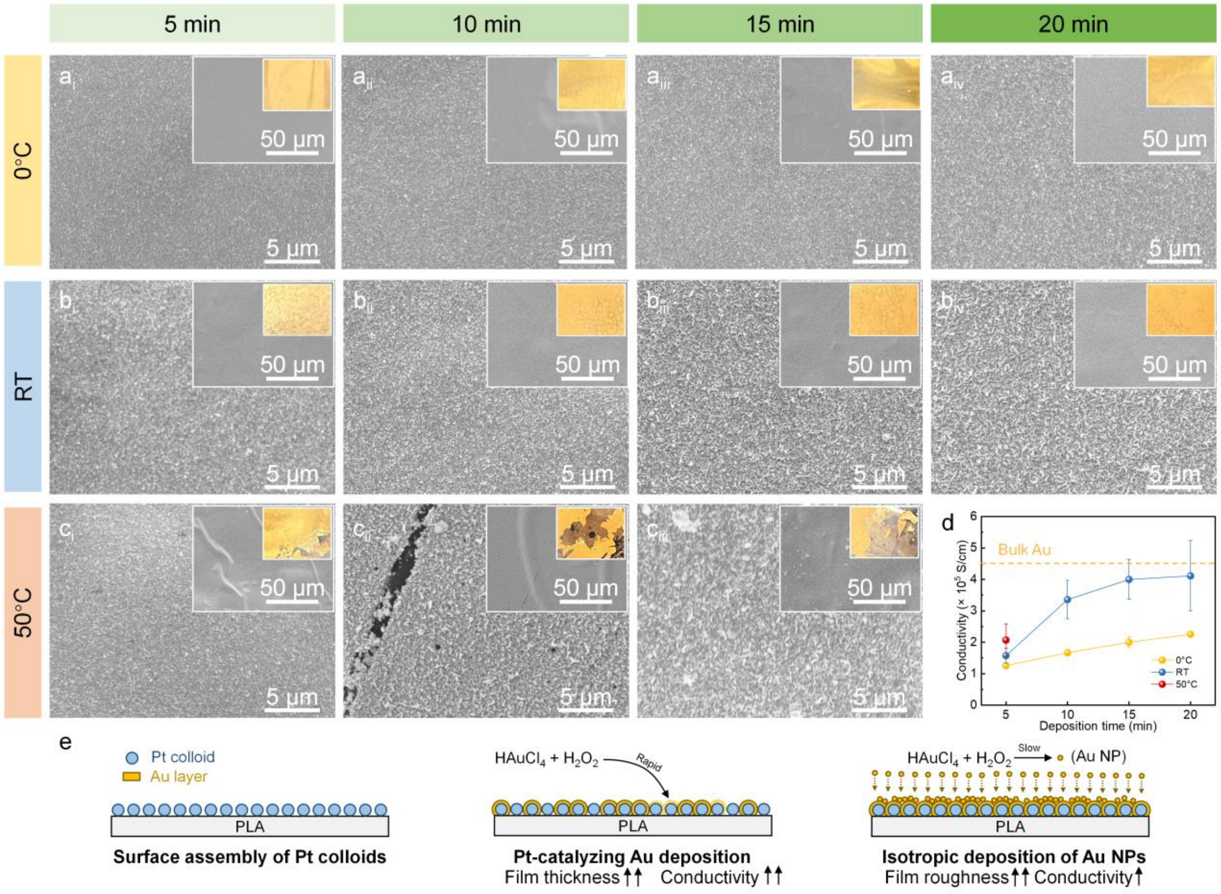

Figure S4. Surface observation of electroless plated PLA/Au surface. Au deposited at (a_i-a_{iv}) 0°C, (b_i-b_{iv}) RT, and (c_i-c_{iii}) 50°C for various time. (d) Conductivity of the deposited Au coating. The samples deposited at 50°C for 10 and 15 min exhibited cracks, and conductivity was not recorded. (e) Schematics of Au electroless plating mechanism. The Pt catalyzing deposition exhibits a deposition procedure to form a uniform layer, while the high deposition rate under high temperature increases the surface roughness.

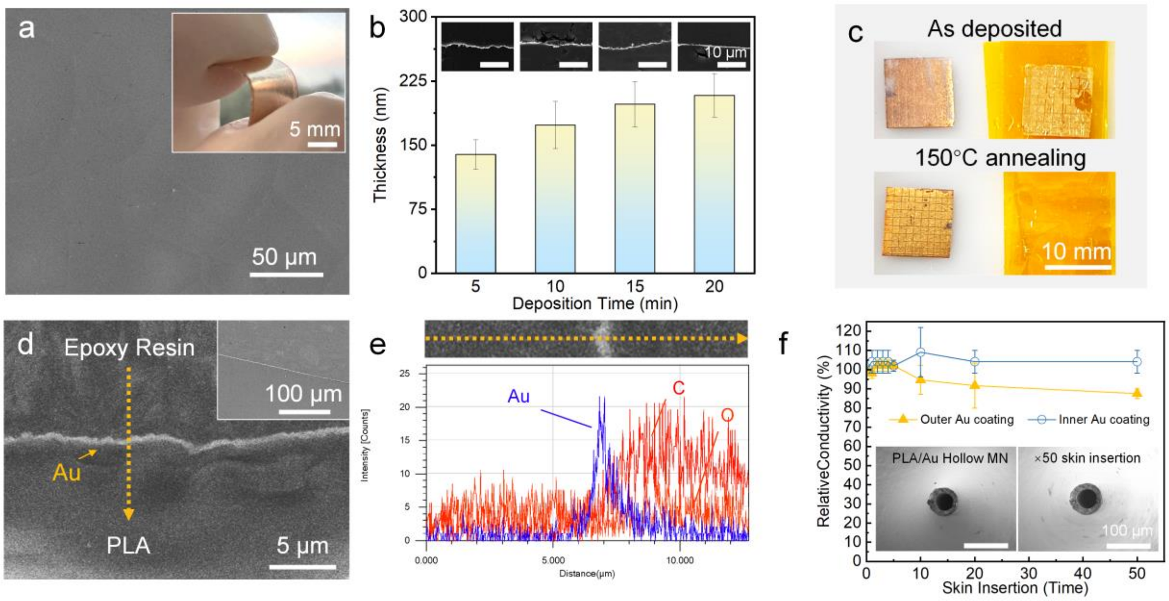

Figure S5. Characterization of PLA/Au electrodes. (a) SEM and optical images of flat PLA/Au electrode. (b) Relationship between thickness and deposition time. (c) Enhanced coating adhesion force after annealing at 150 °C. (d) Cross-sectional SEM images, and (e) line scanning EDS results of the electrodes. (f) Characterization of PLA/Au hollow MN after inserting into porcine skin for 50 times.

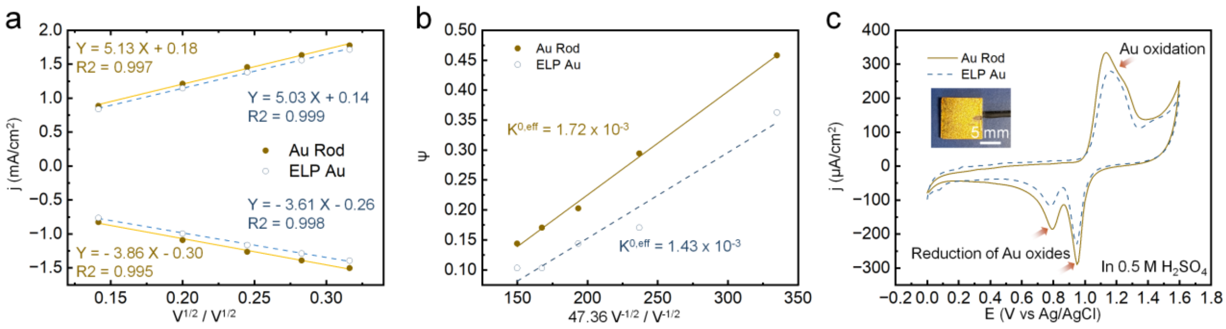

Figure S6. Electrochemical performance of electroless plated PLA/Au electrodes. (a) Relationship between the peak current and the scan rate. (b) Calculation of the effective rate constant ($k^{0,eff}$) values for Au rod and PLA/Au electrodes. (c) Comparison of CV curves for Au rod and PLA/Au electrodes in 0.5 M H_2SO_4.

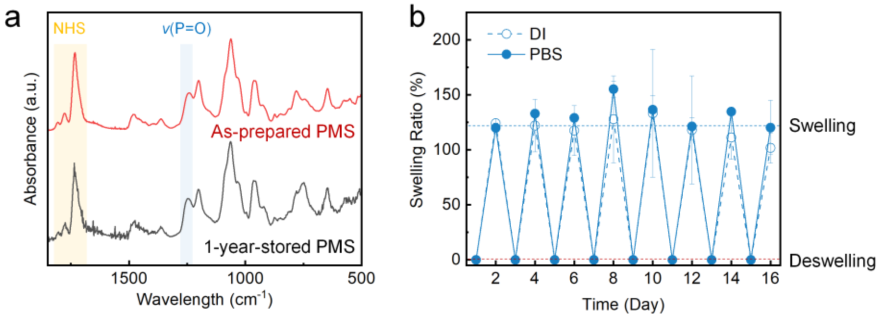

Figure S7. Characterization of PMS and PMMFc. (a) FT-IR analysis comparing freshly prepared PMS with PMS stored at -30 °C for 1 y. (b) Swelling and de-swelling experiment of PMMFc over 2 w.

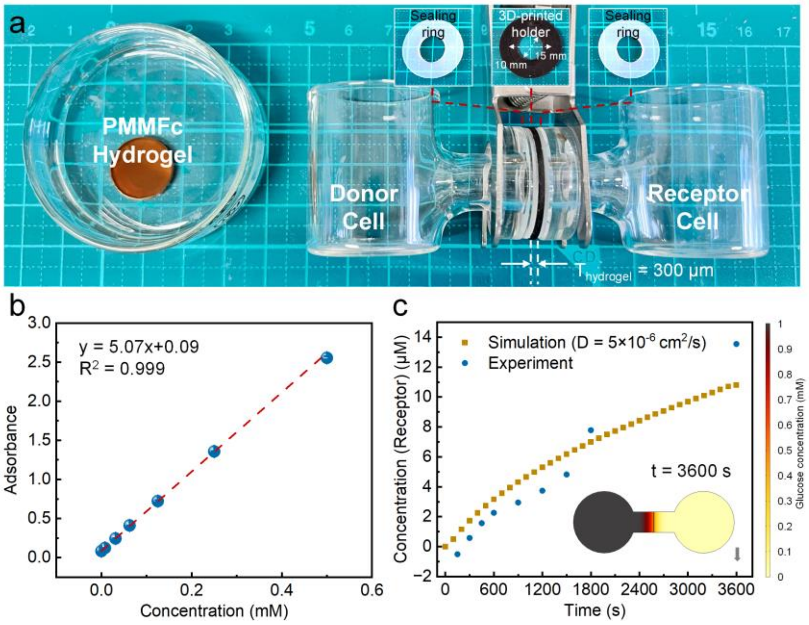

Figure S8. Glucose diffusion coefficient measurement of PMMFc hydrogel. (a) Experimental setup showing PMMFc hydrogel membrane and side-to-side Frantz cell. The 3D-printed holder is used to fix the PMMFc in the joint with a diameter of 10 mm. (b) Calibration curve depicting absorbance against glucose concentration, following the protocol of the commercialized enzyme colorimetric assay kit (Dojindo, Japan). (c) Simulation results verifying the experimentally obtained glucose diffusivity.

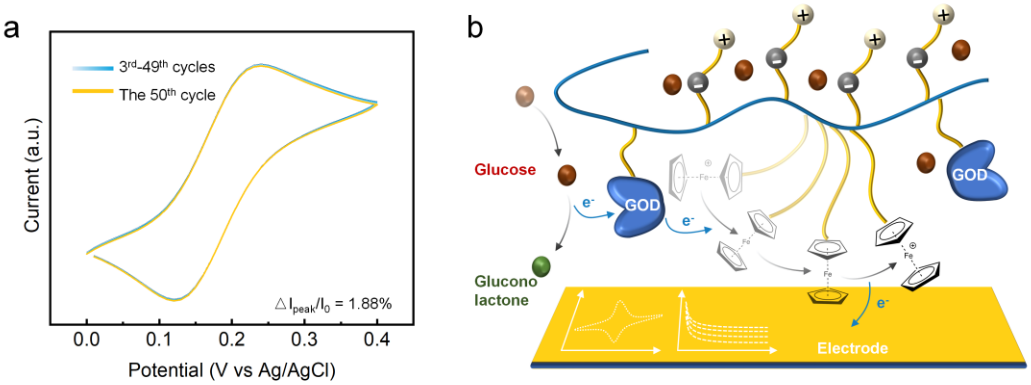

Figure S9. Stability evaluation of PMMFc-GOD hydrogel on PLA/Au electrode. (a) Continuous CV cycles measured in PBS at a scan rate of 100 mV/s. (b) Schematic illustration of the electron transfer process from the enzyme to the electrode via ferrocene moieties immobilized in the zwitterionic polymer hydrogel matrix.

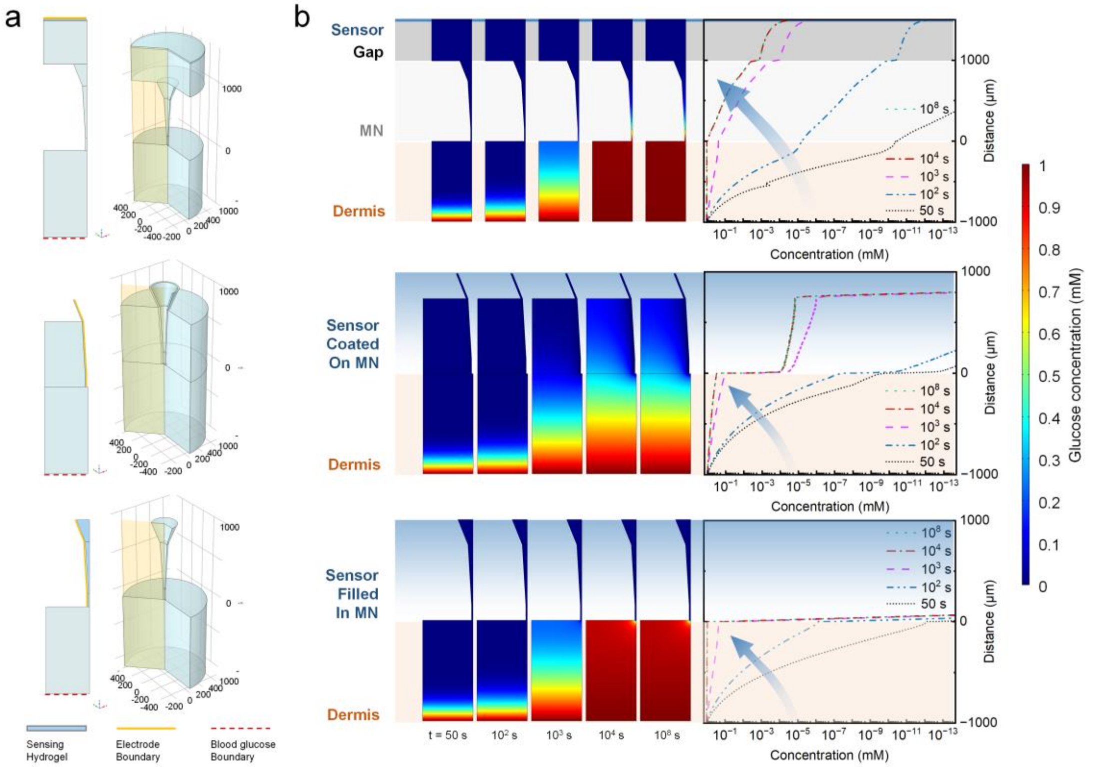

Figure S10. Numerical simulation on glucose diffusion using COMSOL. (a) Three 2D-axisymmetric models used to simulate three hydrogel-assembly designs of off-skin, on MN, and in MN. (b) Set the initial concentration value to 0 and fix the bottom boundary concentration to 1mM. 2D screenshot of the diffusion trend of glucose concentration and the projection of the concentration average in the xy plane on the z-axis

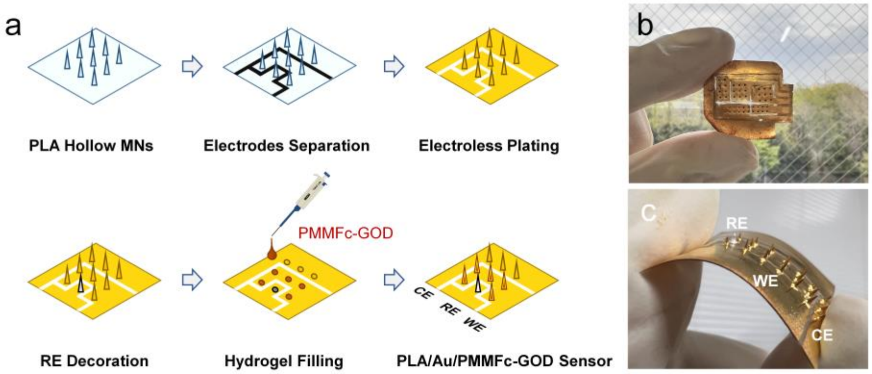

Figure S11. Fabrication procedures of PLA/Au/PMMFc-GOD sensor. (a) Schematics of the circuitization route for PLA/Au electrodes. (b) Electrically isolated 5-partitioned 44-microneedle array sensor. (c) Typical PLA/Au/PMMFc-GOD 9-MN sensor batch prepared with a design featuring 1 RE, 5 WEs, and 3 CEs.

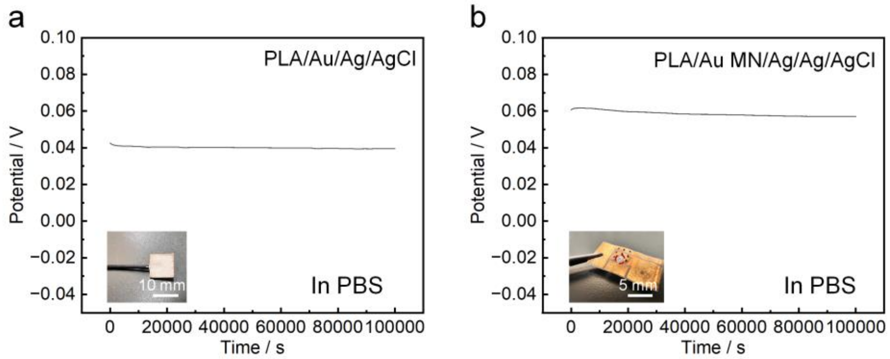

Figure S12. Long-term stability of the Ag/AgCl reference electrode on (a) PLA/Au and (b) PLA/Au MN. The experiment was conducted in comparison to a standard Ag/AgCl external reference electrode.

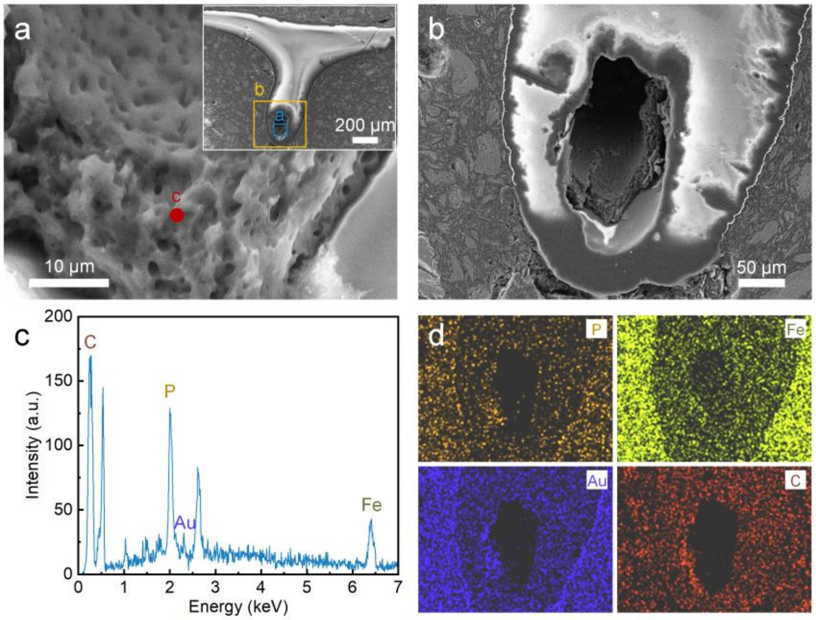

Figure S13. Morphologic and elemental characterization of PLA/Au/PMMFc-GOD MN sensor. (a) Porous hydrogel tightly connects to the sidewall. (b) Zoomed-in region of the hollow channel. Elemental analysis results obtained through EDS for (c) point scanning of (a) and (d) area scanning of (b).

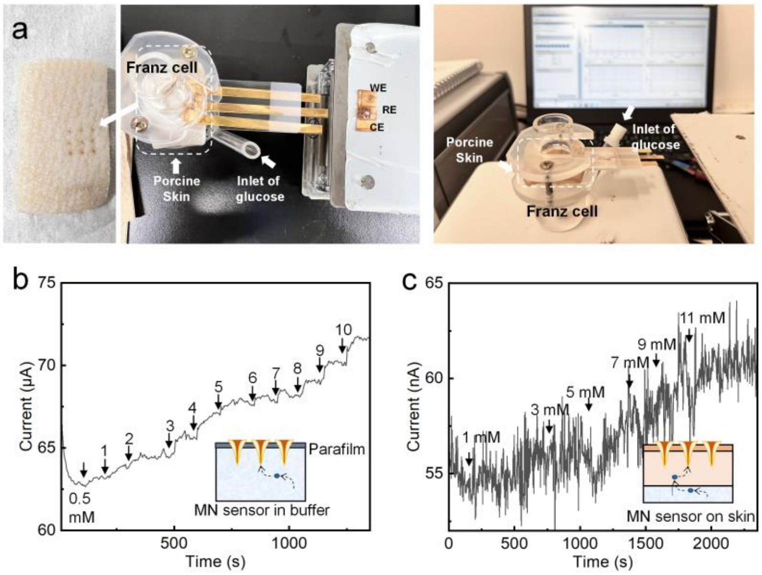

Figure S14. Experimental setups and the chronoamperometric measurement of glucose. (a) Experimental setup of 3×3 PLA/Au/PMMFc-GOD MN sensor (with 1 RE, 3 CEs, and 5 WEs) on the porcine skin. The left picture exhibits the successful insertion of the MN sensor. The glucose level can be adjusted through the inlet. The chronoamperometric response of glucose on the Franz diffusion cell sealed with (b) parafilm and (c) 4-mm-thick porcine skin.

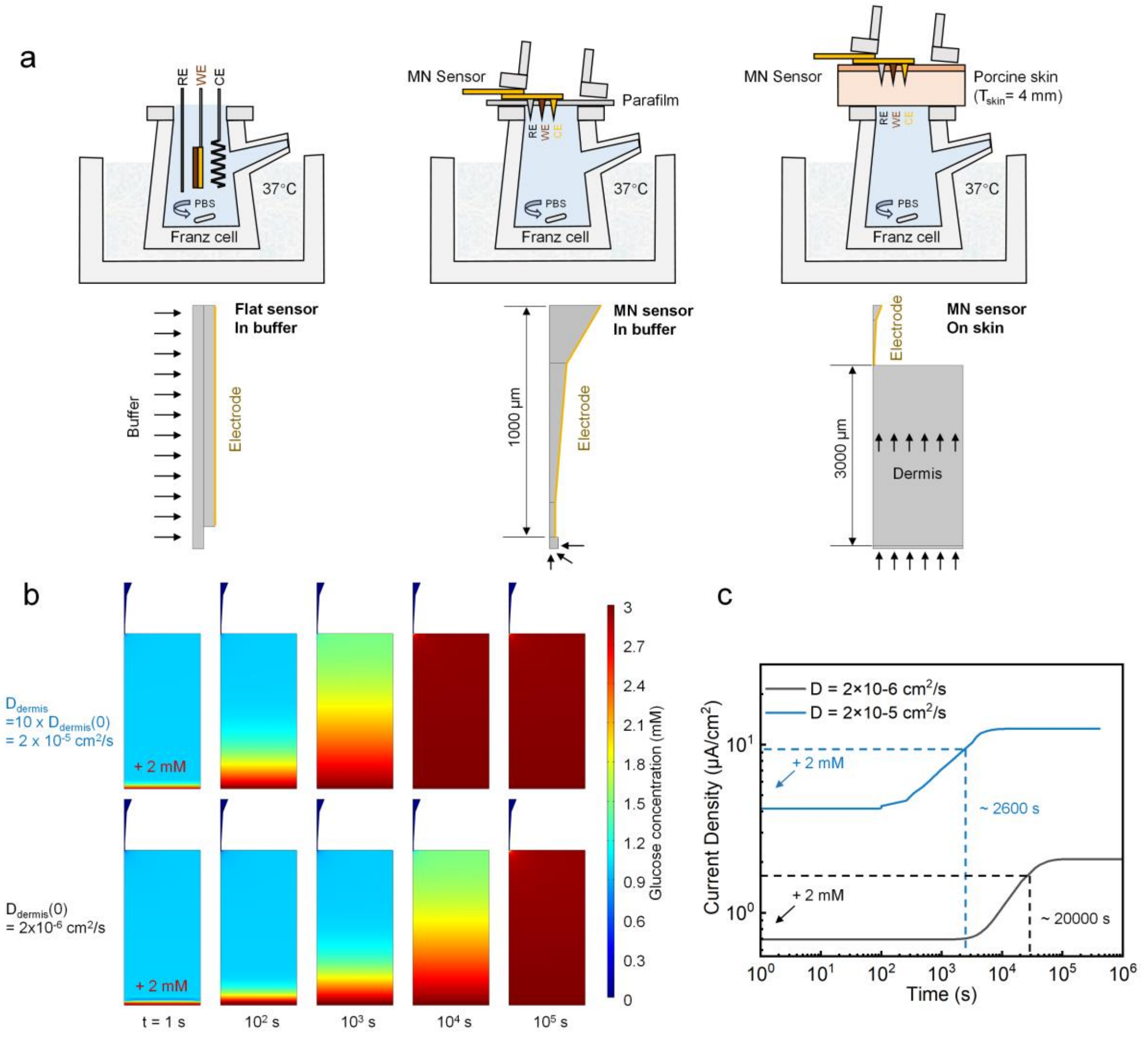

Figure S15. Experimental setups and numerical simulation on glucose diffusion. (a) Schematic diagrams and simulation models representing three distinct experimental setups. (b) Two-dimensional projections illustrating the glucose concentration and (c) simulated chronoamperometric responses observed when the glucose concentration at the dermis increases to 2 mM, based on the MN-sensor-on-skin model under two different diffusivity conditions.

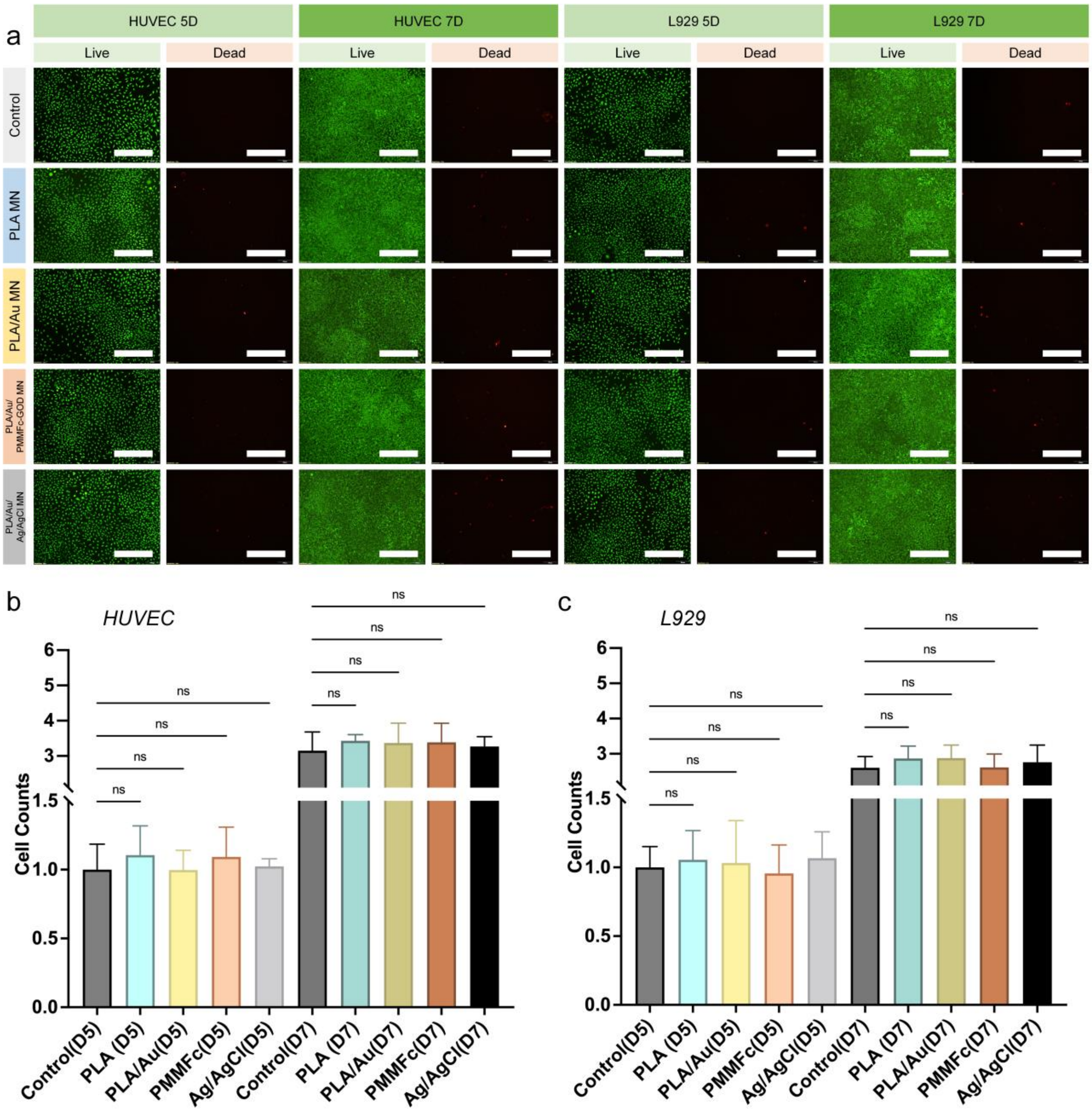

Figure S16. Biocompatibility evaluation of MN electrodes. (a) Live/Dead assay results for HUVEC and L929 cells co-cultured with different MN electrode treatments over 5 and 7 days. The treatments include Control, PLA MN, PLA/Au MN, PLA/Au/PMMFc-GOD MN, and PLA/Au/Ag/AgCl MN. Green fluorescence indicates live cells, and red fluorescence indicates dead cells. Scale bars represent 500 μm. (b) Quantitative analysis of HUVEC cell viability and (c) L929 cell viability co-incubated with various MN electrodes over 5 and 7 days. Cell counts are presented as ratios relative to the control (D5).

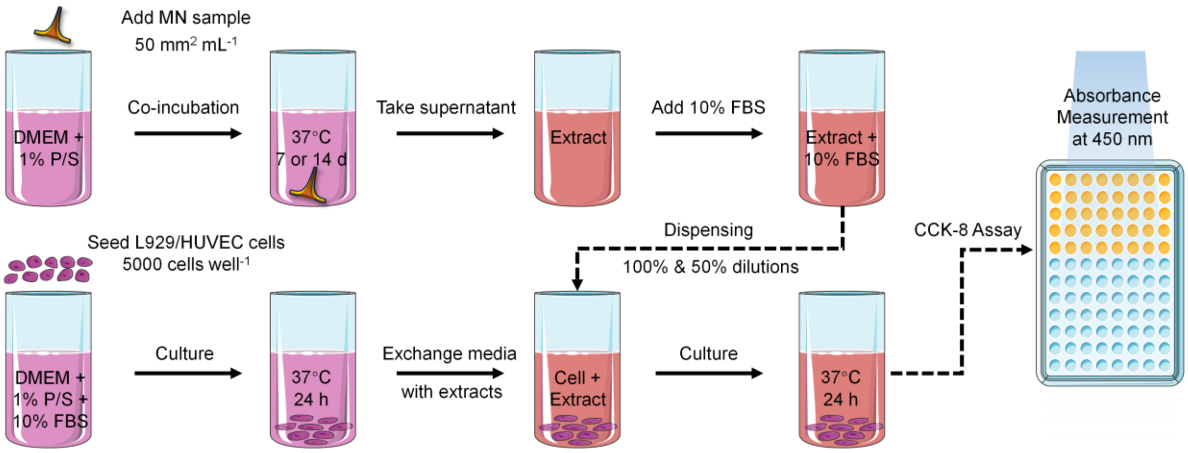

Figure S17. Schematic diagrams representing the measurement of cytotoxicity of MN electrodes using extraction medium and CCK-8 assay.

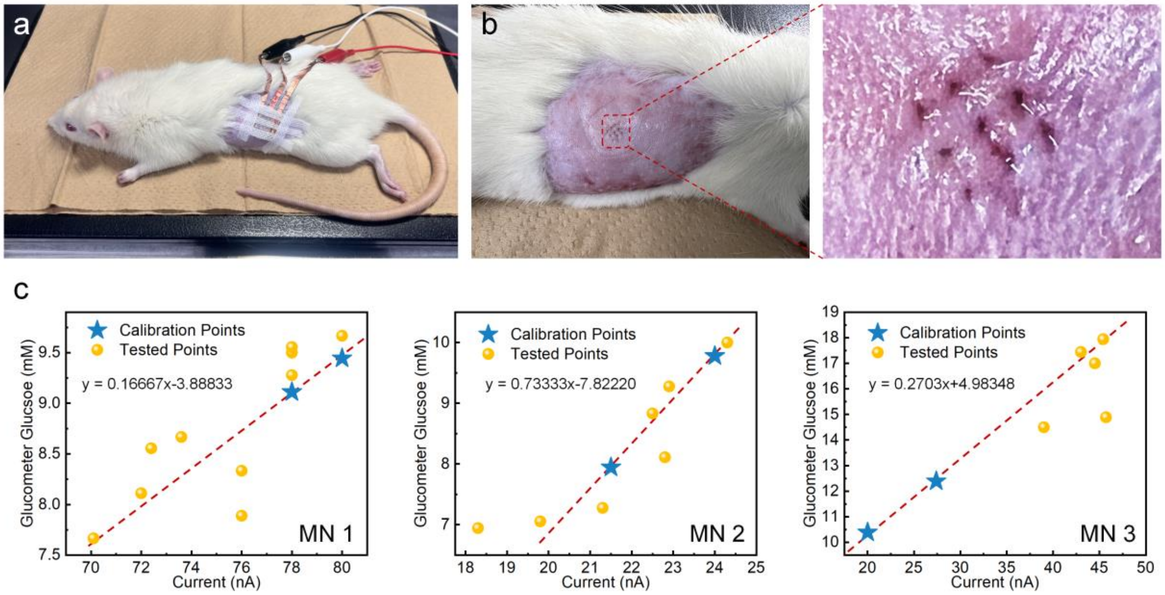

Figure S18. *In vivo* glucose monitoring performance of PLA/Au/PMMFc-GOD MN sensor. (a) Photograph of the MN sensor secured on the lateral abdomen of a rat and connected to an external potentiostat. (b) Observation of the rat skin after insertion with the MN sensor. (c) Two-point calibration curves of the MN sensor.

Table S1. List of band assignments for FTIR spectra.

Wavenumber (cm^{-1})	Assignments	
1255	ν(P=O)	P=O stretch
1525–1615	δ(N-H)	N-H bending
	ν_{asym}(COO-)	O-C=O antisymmetric stretch
1732	ν_{asym}(imidyl C=O)	NHS ester C=O antisymmetric stretch
1777	ν_{sym}(imidyl C=O)	NHS ester C=O symmetric stretch
1805	ν(ester C=O)	NHS ester carbonyl stretch

Table S2. Summary of EIS fitting parameters for different samples.

	PLA/Au in PBS	PLA/Au in PBS + 50 mg/ml BSA	PLA/Au/PMMFc-GOD in PBS	PLA/Au/PMMFc-GOD in PBS + 50 mg/ml BSA
Rs ($\Omega \cdot cm^2$)	3.21E+01	3.44E+01	1.95E+01	3.90E+01
CPE-Yo ($S \cdot cm^{-2} \cdot s^n$)	4.95E-05	2.97E-05	3.31E-05	3.08E-05
CPE-n	8.76E-01	9.11E-01	9.00E-01	8.97E-01
Rct ($\Omega \cdot cm^2$)	1.23E+05	2.23E+05	8.05E+03	7.80E+03
W ($S \cdot cm^{-2} \cdot s^{0.5}$)	1.85E-04	8.67E-05	9.08E-04	9.08E-04

Table S3. Parameters used in COMSOL simulation.

Symbol	Default Value	Description
h_{dermis}	1000E-6[m]	Dermis thickness
w_{dermis}	1000E-6[m]	Dermis width
w_{tip}[a)]	15E-6[m]	Tip width
h_{tip}[a)]	1000E-6[m]	Tip length
w_{base}[a)]	150E-6[m]	Tip base width
k_1	1E3[1/mM/s]	Reaction constant 1
k_2	1E2[1/s]	Reaction constant 2
k_3	1E5[1/s]	Reaction constant 3
k_4	3E6[1/mM/s]	Reaction constant 4
k_5	2E4[1/s]	Reaction constant 5
k_6	1E5[1/s]	Reaction constant 6
$c_{glucose}$	1[mM]	Glucose concentration
$c_{mediator}$	5[mM]	Total mediator concentration
c_{enzyme}	0.1[mM]	Total enzyme concentration
D_{PMMFc}[a)]	5E-10[m^2/s]	Glucose diffusivity (hydrogel)
D_{dermis}	2E-10[m^2/s]	Glucose diffusivity (dermis)
$D_{mediator}$	1E-10[m^2/s]	Mediator diffusivity
D_{enzyme}	1E-15[m^2/s]	Enzyme diffusivity
k_0	1.2E4[m/s]	Heterogeneous rate constant
E_0[a)]	0.18[V]	Formal potential of the reaction
E_{ap}[a)]	0.24[V]	Applied potential
a	0.5	Transfer coefficient
R_0	8.314[J/K·mol]	Ideal gas constant
F	96485[C/mol]	Faraday constant

[a)] Tested value
